# Mechanical Properties of Iron Tailing Sand Grout Sleeve Joints and Force Analysis

**DOI:** 10.3390/ma17194900

**Published:** 2024-10-06

**Authors:** Fuyin Guo, Jiahao Wang, Lin Zhao, Pan Guo, Dong Wei, Yuanxun Zheng, Zhe Zhang, Enfeng Deng

**Affiliations:** 1 Henan Zhonggong Design & Research Group Co., Ltd., Zhengzhou 450018, China; 2 School of Water Conservancy and Transportation, Zhengzhou University, Zhengzhou 450001, China; 3 Yellow River Laboratory, Zhengzhou University, Zhengzhou 450001, China; 4 Hengzhongda Construction Co., Ltd., Shanghai 200062, China; 5 State Key Laboratory of Tunnel Boring Machine and Intelligent Operations, Zhengzhou 450001, China; 6 School of Civil Engineering, Zhengzhou University, Zhengzhou 450001, China

**Keywords:** iron tailing sands, grouting sleeves, mechanical properties, internal stresses in joints

## Abstract

In this paper, the mechanical properties and internal stress condition of the reinforcing bar sleeve connectors with ferro-tailed mineral sand cementitious grout as filler material were analyzed as research objects. Firstly, an experimental study was carried out on the reinforcing bar sleeve connectors of iron tailing sand grout with a 40% substitution rate of mechanism sand to analyze the mechanical properties of different grout types, age, and reinforcement diameters under unidirectional tensile, high stress, and large deformation of repeated tensile and compressive stresses. Next, five groups of sleeve joints with different anchorage lengths were set up for unidirectional tensile tests. The results show that, with the decrease of the diameter of the reinforcement, the grip force and bond strength of the iron tailing sand grout on the internal reinforcement gradually increase. Under conditions of large deformation and high stress due to repeated tensile loading, the residual deformation and total elongation of iron tailing sand grout sleeve joints are satisfactory. Additionally, the restraining anchorage effect of iron tailing sand grout in the end section is small. The utilization rate and integrity of iron tailing sand grout in the initial anchorage section are better.

## 1. Introduction

With the urgent need for green and sustainable development, the advancement of intelligent buildings, and the improvement of human expectations for the living environment, the technology of prefabricated buildings has been gradually improved [1]. In the prefabricated building, the connection of the node structure is the key problem, the steel bar is the main connecting link, and the connection mode of the steel bar has become the core technology of the node connection. At present, the main connection methods of steel bars are mechanical connection, lashing, lap, and welding. The traditional lashing, lap, and welding technology has a large amount of on-site construction, and the connection quality is greatly affected by various factors. Mechanical connection technology is a new type of steel bar connection process by pouring high-strength cementitious materials, so as to realize the extrusion and occlusion between cementitious materials, steel bars, and sleeves. Among the mechanical connection methods, the grouting sleeve connection method has been widely used in practical engineering because of its advantages of economy, short construction period, and fast construction speed [2,3,4,5]. This connection can be made possible by grouting reinforcement to achieve force transfer between the members to ensure the integrity and continuity of the prefabricated structure [6,7]. On the other hand, with the increasing global energy shortage and environmental pollution, green, low-carbon, energy-saving, and environmental protection have become the basic requirements for the economic development of countries around the world [8]. In recent years, with the innovation of technology and the advances in productivity, China’s mineral industry has flourished, but a large amount of iron ore tailings have also been produced in the separation process of iron minerals obtained from ores [9]. Iron tailings are generally treated by direct landfill, which not only causes serious pollution to the surrounding ecological environment but also further leads to a waste of land resources. Therefore, the utilization of solid waste for iron tailings is of great significance in the current situation of lack of resources in China. At present, scholars at home and abroad [10,11,12,13,14,15,16] have extensively studied the use of iron tailings as a partial replacement for aggregates to prepare concrete materials and cement-based grouts in solid waste utilization. The results of the study consistently show that the incorporation of iron tailings has little effect on the performance of grouting material and has good technical feasibility. Therefore, the use of iron tailing sand to replace manufactured sand to prepare cement-based grout has certain research value.

However, the grouting sleeve connection also has the problems of low strength, poor toughness, and insufficient durability. Among them, the bonding and anchoring performance of grouting steel bars has a significant effect on the service performance of steel reinforcement sleeves [17,18,19,20]. At present, some experts and scholars have carried out a series of studies on the mechanical properties of steel reinforcement sleeves. Ling et al. [21] conducted an experimental study of four different types of grouting sleeves and found that changing the shape of the sleeve (e.g., reducing the inner diameter of the sleeve) can improve the ultimate bearing capacity of the sleeve joint. Liu et al. [22] fabricated 15 grouting sleeve joint specimens to study the failure process of the sleeve joint under uniaxial tensile load. The results show that an anchorage length greater than 7 d is necessary to prevent sliding failure at the interface between the grouting and the reinforcement. Han et al. [23] designed 24 grouting sleeve joints to study the effect of grouting material strength on the bond strength of sleeve joints under uniaxial tensile loading. The results show that the relative displacement between the reinforcement and the grouting material is generated after the hardening stage of the tensile process, revealing the relationship between the strength of the grouting material and the bond strength. Compared to carbon steel sleeves, ductile iron sleeves exhibit better adhesion properties during the stretching process. Zhang et al. [24] performed unidirectional tensile tests on 12 semi-grouting specimens and 12 single bars at different temperatures to analyze the adhesion characteristics of the semi-grouting sleeve connection at high temperatures. The results show that when the temperature increases, the fracture position and failure mode of the rebar of the joint may change. The anchorage length is sufficient at room or low temperature but becomes insufficient at 600 °C. Lei et al. [25] performed tensile tests on 24 specimens to investigate the mechanical properties of grouting sleeve splices. The results show that the anchorage length of the rebar using ultra-high-performance grouting materials can be shorter than recommended by the design standard. The anchorage length and eccentricity of the steel bar have an important impact on the mechanical properties of the grouting sleeve splicing. Zheng et al. [26] studied the mechanical properties of 24 pre-designed sleeve connections with insufficient grouting defects and considered the case of repairing the sleeve connection by refilling the sleeve with insufficient grouting material. The results show that, with the decrease in the anchorage length of the rebar, the failure mode of the defective specimen may change from tensile fracture of the rebar to the interfacial bond-slip failure of the rebar. With the exception of deformation, the repaired sleeve connection has similar mechanical properties to a fully grouted sleeve connection.

From different perspectives, the above scholars explored the influence of factors such as sleeve shape, steel bar diameter, grouting material strength, and high-temperature environment on the mechanical properties of grouting sleeve joints. In order to further explore the performance of iron tailing grout in steel bar sleeve connectors, 78 steel sleeve connecting joints were made by using iron tailings grouting with the best substitution rate (40%). The effect of iron tailing grout on the strength and deformation properties of sleeve joints was explored. Different parameters such as anchorage length, steel bar diameter, age, and protective layer thickness were set to conduct unidirectional tensile, large deformation, and high-stress repeated tensile and compression tests of sleeve joints. The influence of different parameters on the performance of joints was analyzed, and the feasibility of using new iron tailing cement-based grout for sleeve joints was verified. At the same time, by attaching strain gauges between the surface of the sleeve and the anchor steel bar, the stress distribution law between the surface of the sleeve and the interface between the steel bar and the grouting material was studied. The internal force changes in the cement-based grouting material containing iron tailings when the load was applied were further explored, and the anchoring effect of the iron tailing grouting sleeve was verified.

## 2. Experimental Design

### 2.1. Performance Requirements of Sleeve Joint

Evaluating the mechanical properties of iron tailing sand grout sleeve joints through relevant tests is the key focus of this section. General Technical Specification for Mechanical Connection of Reinforcing Steel Bars (JGJ107-2016) [27] classifies mechanical connection joints into the following three grades according to the ultimate tensile strength of the joints (Table 1):

At the same time, all levels of joints in the uniaxial tensile test and high-stress repeated tensile and large-deformation repeated tensile tests under the deformation performance should be consistent with the provisions of Table 2.

According to the relevant requirements in the “Steel Bar Sleeve Grouting Connection Application Technical Specification (JGJ355-2015)” [28], when the grout composition is altered, the grouting sleeve joints should be subjected to joint type inspection. Therefore, the reinforcing steel sleeve grouting connection joints should also comply with the following provisions:

(1) The yield strength of the grouting sleeve joint shall not be less than the standard value of the yield strength of the connecting steel bar;
(2) Grouting sleeve joints, during the uniaxial tensile, high-stress repeated tensile, and large-deformation repeated tensile tests, must withstand a tensile force 1.15 times the standard tensile load value of the connecting steel bar without failure.

### 2.2. Specimen Design

#### 2.2.1. Grout

In this paper, on the basis of existing research [29], we selected the replacement rate of 40% iron tailing sand grout and benchmark manufactured sand grout. The mechanical properties are detailed in Table 3.

#### 2.2.2. Sleeve

**Figure 1:**
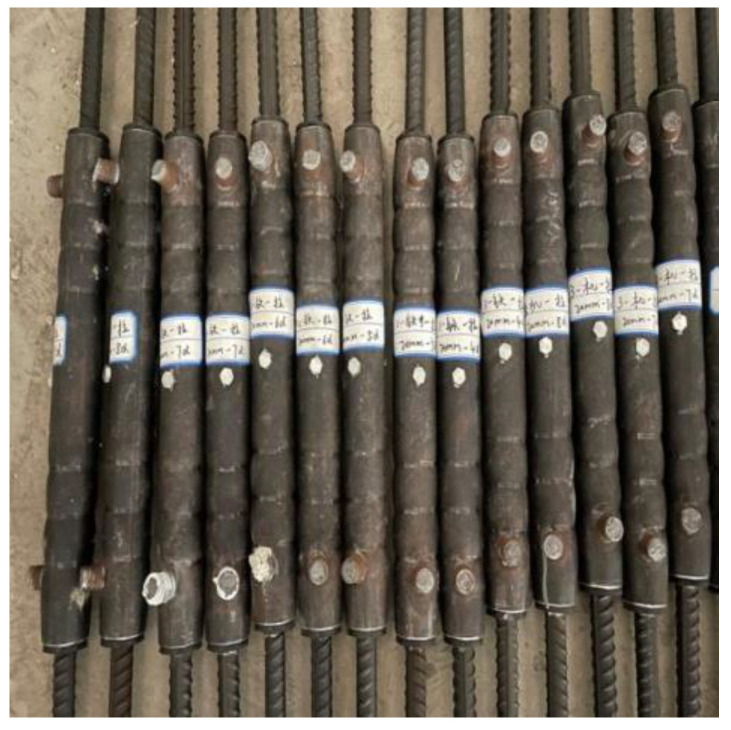
Steel full grouting sleeve.

**Figure 2:**
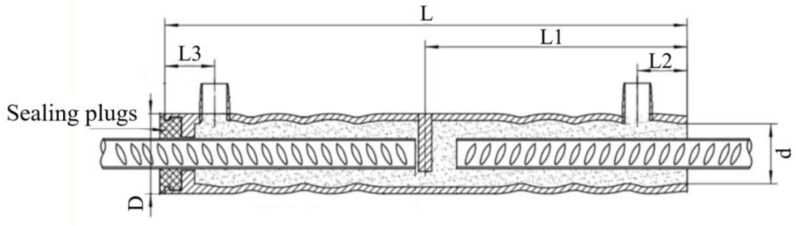
Schematic diagram of steel full grouting sleeve.

Sleeves were made of GTQ4J-type full grouting sleeves, made of carbon steel, see Figure 1. There are five types of sleeve models, corresponding to five different diameters of rebar, whose schematic diagrams and dimensions are shown in Figure 2 and Table 4. D is the outer diameter of the sleeve, and d is the inner diameter of the sleeve; L is the total length of the sleeve; L1 is the length of the non-grouted end; L2 is the distance from the outlet hole to the port; and L3 is the distance from the grout hole to the port.

#### 2.2.3. Reinforcing Steel

**Figure 3:**
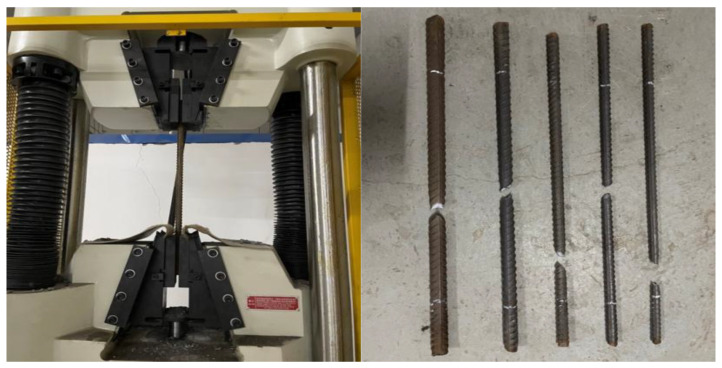
Reinforcing steel material property test (16, 18, 20, 25, 28 mm).

HRB400E rebar was used, with five diameters of 16 mm, 18 mm, 20 mm, 25 mm, and 28 mm. Use the universal testing machine to carry out the material properties of the steel bar test, such as Figure 3, five diameters of the yield strength and tensile strength of the steel bar are shown in Table 5.

### 2.3. Experimental Parameter Design

Two grout types, five anchorage lengths, five bar diameters, two grouting material ages, and rebar offsets (protective layer thickness) were set as parameters. A total of 26 groups, each containing three joint specimens, resulted in a total of 78 specimens. There are three loading regimes: uniaxial tensile, repeated high-stress pulling and pressing, and repeated pulling and pressing with large deformation. The test results and key performance indicators are averaged. Specific parameter settings and specimen numbering are shown in Table 6 and Table 7.

### 2.4. Specimen Processing and Fabrication

**Figure 4:**
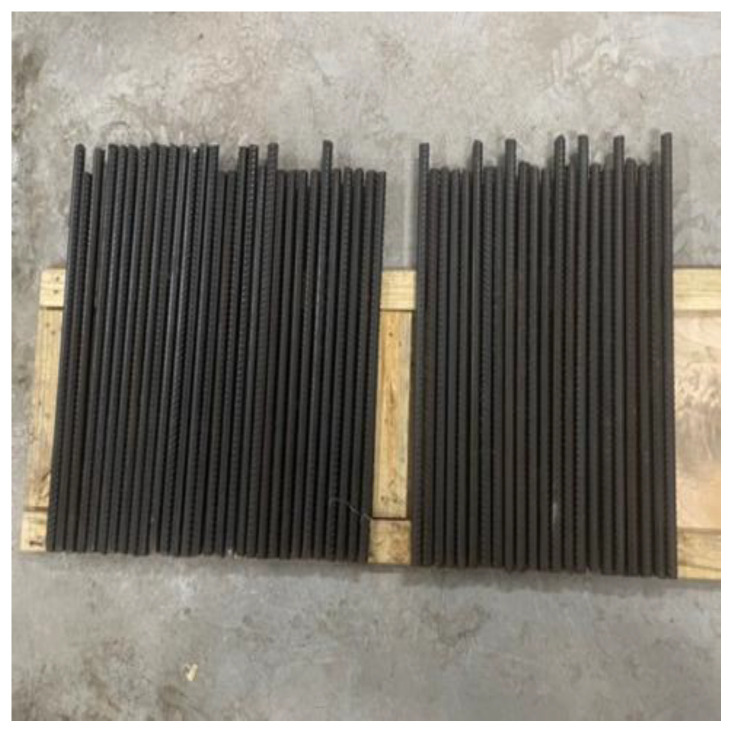
Reinforcement bars after descaling.

**Figure 5:**
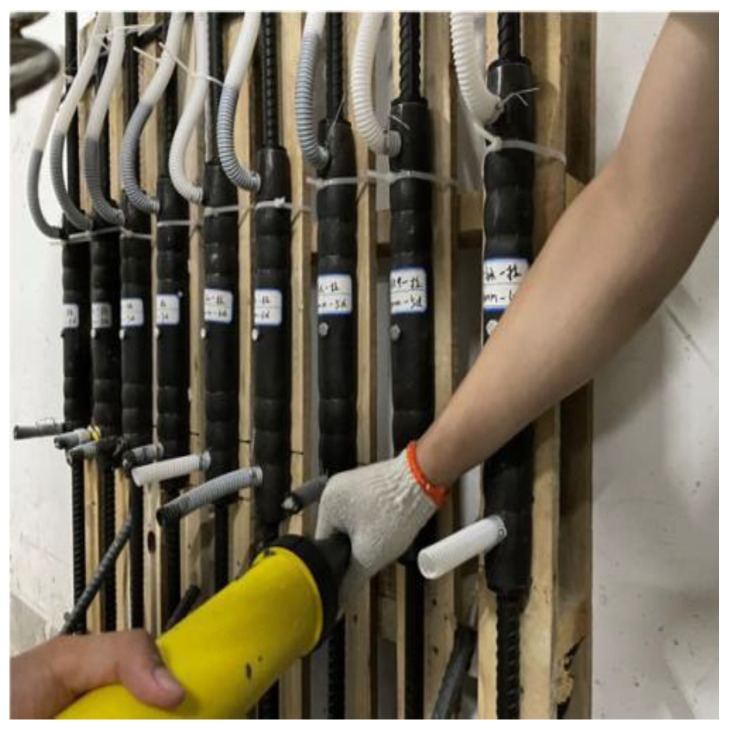
Grouting process.

**Figure 6:**
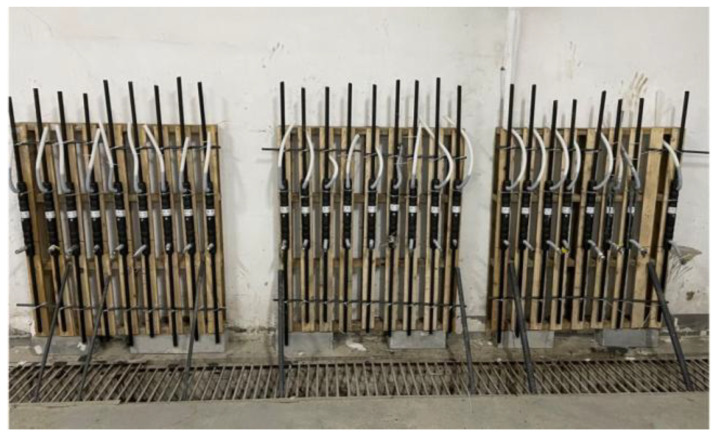
Partial specimen.

In this test, a fully grouted sleeve was used for the connection. Firstly, the steel bars were descaled and marked with a white marker according to the designed anchorage length. In order to prevent the rebar from being eccentric after insertion of the sleeve, the sleeve was fixed vertically on a homemade bracket with tie wraps and pads to ensure that the rebar position was centered. A hand-held grouting gun was used to inject the configured and mixed grout from the lower grouting port. In order to ensure that the grout is full, a hose with a height of more than ten centimeters above the end of the sleeve was connected to the outlet. Part of the production process is shown in Figure 4, Figure 5 and Figure 6. The finished sleeve was maintained on the bracket for two days, then removed to continue to be maintained at room temperature for a total of 28 days after the grout had completely solidified.

## 3. Specimen Destruction Mode

**Figure 7:**
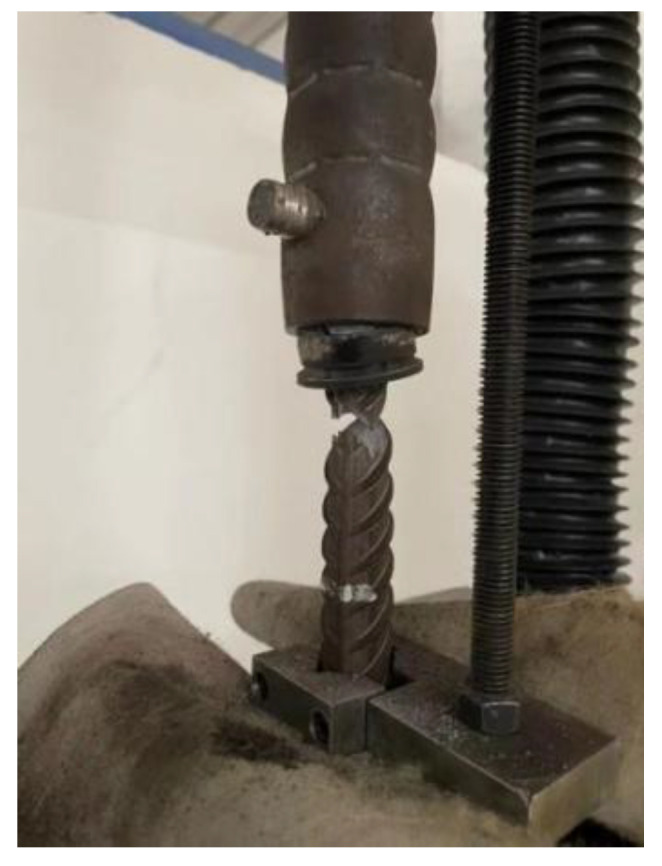
Rebar pull-off.

**Figure 8:**
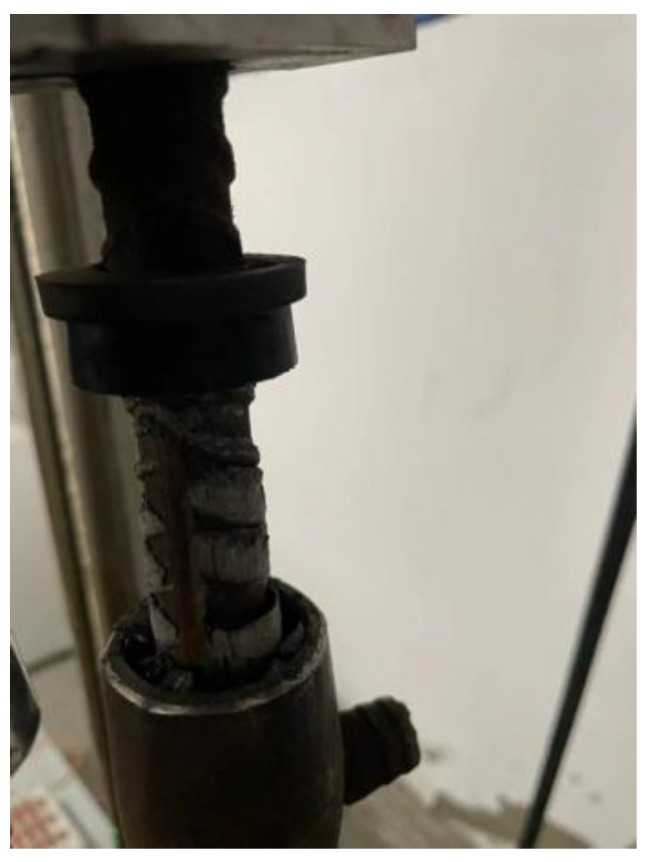
Rebar pull-out.

**Figure 9:**
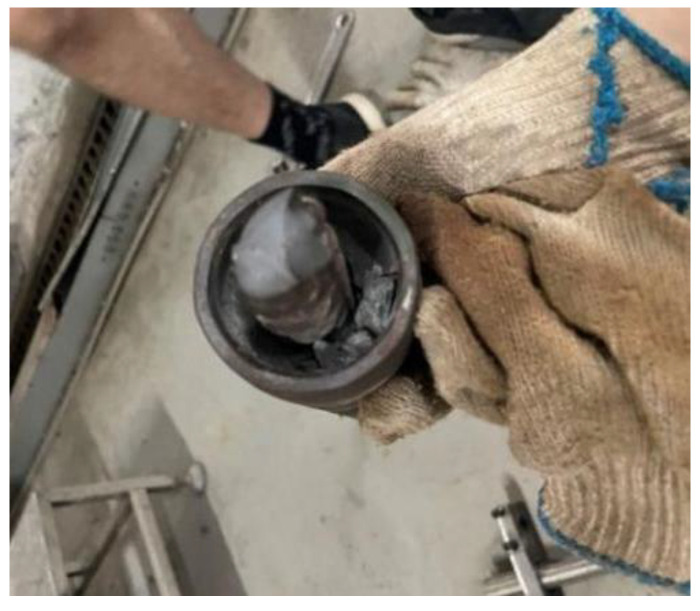
Grout crumbles.

As shown in Figure 7, Figure 8 and Figure 9, according to the experimental study, there are two damage modes of sleeve joints: pulling out of the steel bar and pulling off of the steel bar outside the sleeve. Theoretically analyzed, the damage modes of fully grouted sleeves may also occur such as sleeve failure and grout pulling out from the sleeve. Therefore, the strength of the iron tailing sand grout, the ultimate strength of the steel bar, the strength of the sleeve, the bond strength of the steel bar to the iron tailing sand grout, and the bond strength of the iron tailing sand grout to the sleeve all have an impact on the use of the grouted sleeve. The fact that the remaining damage patterns did not occur in the tests suggests that the form of damage to the sleeve joints in the tests depended only on the ultimate strength of the steel bar and the anchorage strength between the iron tailing sand grout and the anchoring steel bar.

(1) Damage of steel bar pulling off

When the anchorage strength between the iron tailing sand grout and the steel bar is greater than the ultimate tensile strength of the steel bar, the steel bar will be pulled off. Specimens in the elastic phase and yield phase will produce intermittent sounds, as the internal steel bar surface and the iron tailing sand grout between them experience changes in mechanical bite force. The external steel bar becomes significantly thinner, and the displacement gauge bracket will be dislodged as a result, but there is still a certain bond strength between the anchoring steel bar and the grout. During continued loading, there is a slight dislocation of the end grout; with further loading to destruction, the steel bar continues to become thinner, and necking occurs rapidly at a location of either the external upper or the external lower steel bar, which is then accompanied by a loud rupture sound, and the steel bar is pulled off from the outside of the socket.

(2) Steel bar pull-out damage

When the anchorage strength between the iron tailing sand grout and the steel bar is less than the ultimate tensile strength of the steel bar, a steel bar pull-out will occur. For all the specimens with rebar pull-out damage, rebar pull-out occurred in the strengthening stage, and most of the failures were caused by insufficient anchorage length. During the strengthening phase, the load continues to rise but does not reach the ultimate load for pull-out, often accompanied by a distinct sound and a steep drop in strength. This is followed by a residual phase in which the steel bar pulls out and is accompanied by the fall of grout crumbs. The displacement gauge brackets likewise fall off as the steel bar thins out.

**Figure 10:**
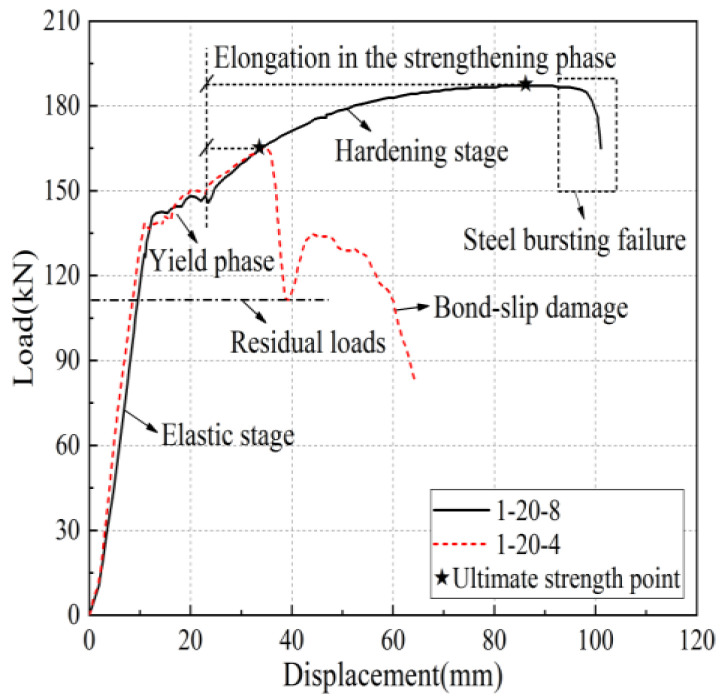
Typical load-displacement curve.

Take specimen 1-20-8 and specimen 1-20-4 as examples, the load-displacement curves of the two are shown in Figure 10, in which the horizontal coordinate is the relative displacement between the upper and lower two rebar fixtures of the universal testing machine. Specimen 1-20-8 shows that the steel bar pulls off, and its curve relationship is more similar to the uniaxial tensile test of a single bar, divided into four stages: elasticity stage, yielding stage, hardening stage, and necking stage. The elasticity stage curve is basically a linear relationship; the specimen stiffness is high, as the iron tailing sand grout and sleeve wall of the shear ring rib and the anchoring steel bar of the transverse rib are mutually extruding. At this stage, the sleeve end of the iron tailing sand grout shows no obvious crack phenomenon; then, it enters the yielding stage. Immediately after entering the ascending stage, the iron tailing sand grout and reinforcing bar anchoring section are fully extruding, and the iron tailing sand grout fracture zone expands, with cracks fully developing, accompanied by some iron tailing sand grout slag falling off. Finally, in the steel bar necking stage, accompanied by a distinct sound, the steel bar is pulled off.

Specimen 1-20-4 exhibits rebar pull-out damage and the elastic and yield sections of the curve almost coincide with that of specimen 1-20-8, but the stiffness is slightly smaller, and it enters the yielding stage a little earlier. After entering the strengthening stage, as the bond strength between the iron tailing sand grout and the anchoring steel bar is less than the tensile strength of the steel bar, the steel bar, together with part of the iron tailing sand grout between the transverse ribs of the anchoring section, is slowly pulled out as the bond is broken. In the pull-out process, the load-displacement curve is wavy, but the joint still shows good ductility and high residual bond strength. There are two main reasons for this: (1) Although the iron tailing sand grout between the cross ribs of the steel bar is crushed; due to the fine aggregate in the iron tailing sand grout, there is no void formation. (2) In the descending stage, the convex ribs on the inner wall of the sleeve impede the slip of the iron tailing sand grout and rebar. The stress accumulated in front of the sleeve began to release, compensating for the loss of some constraint pressure due to the slip surface being hindered, so that the bond stress remains high during the pull-out process.

## 4. Test Results and Analyses

### 4.1. Effect of Anchorage Length on Uniaxial Tensile Properties of Joints

In uniaxial tensile, there are three forms of force between the iron tailing sand grout and the steel bar: the mechanical occlusion force between the protruding transverse ribs of the steel bar and the iron tailing sand grout, the anchorage friction caused by the micro-expansion of the iron tailing sand grout extruding the steel bar, and the chemical bond between the iron tailing sand grout and the surface of the steel bar, which is known as anchoring capacity. Assuming sufficiently high bond strength between iron tailing sand grout and sleeve, the anchorage capacity between iron tailing sand grout and steel bar and the steel bar itself determines the ultimate bearing capacity of the sleeve joint. Therefore, the anchorage length is one of the most important factors influencing the stress performance of sleeve joint specimens.

According to the existing experimental research, this kind of full grouting sleeve joint, when the anchorage length is greater than or equal to 8 d, generally exhibits steel bar pull-off damage. When the anchorage length is less than or equal to 4 d, bond slip damage generally occurs. Therefore, specimens with a rebar diameter of 20 mm were tested with 4 d, 5 d, 6 d, 7 d, and 8 d as variables. The remaining specimens with different rebar diameters were tested with 5 d, 6 d, and 7 d as variables. The specific test parameters are shown in Table 8. Table 9 shows the key performance indicators and test results.

**Figure 11:**
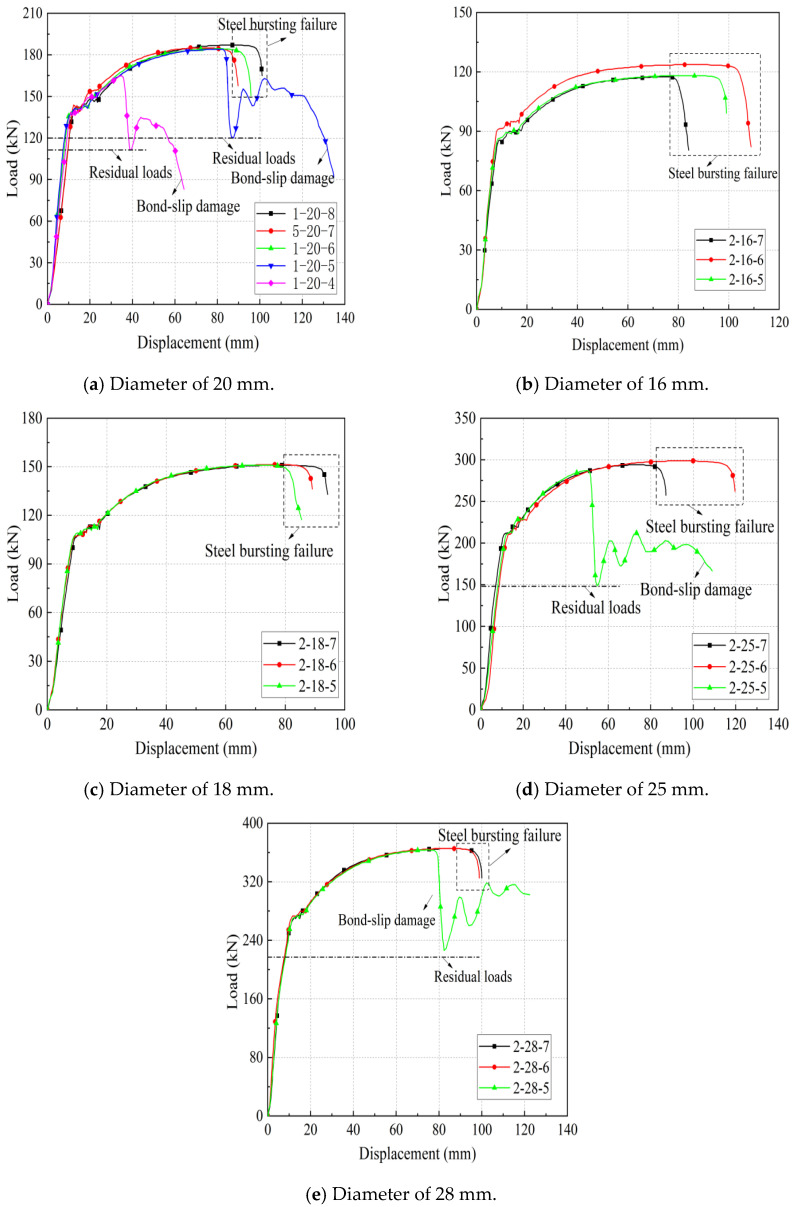
Load displacement curves for different diameters. Different color curves represent different parameter specimens. See Table 8 for specimen information.

As shown in Figure 11, the effect of different steel bar diameters on the mechanical properties of joints was studied. The iron tailing sand grout sleeve joints with a bar diameter of 20 mm were subjected to pull-out damage at anchorage lengths of 4 d and 5 d, indicating that the corresponding effective anchorage lengths under this diameter should be more than 5 d. The ultimate loads were 165.1 kN, 184.7 kN, and 187.2 kN at anchorage lengths of 4 d, 5 d, and 8 d, respectively, and their ultimate loads reached 88.19% and 98.66% at anchorage lengths of 8 d at anchorage lengths of 4 d and 5 d, respectively. Moreover, the residual bond strength of specimen 1-20-5 is significantly higher than that of specimen 1-20-4, indicating that the anchorage length of 5 d is in a critical state. Similarly, for the sleeve joints with diameters of 25 mm and 28 mm, the minimum anchorage length should not be less than 6 d. In addition, the sleeve joints with diameters of 16 mm and 18 mm in this test were damaged by the pull-out of the steel bar.

It is also noted that joint specimens of the same diameter with different anchorage lengths have similar ultimate bearing capacities but different maximum displacements under the steel bar pull-out damage mode. The maximum displacement of grouted sleeve joints during tension consists of the deformation of the steel bar and the sliding displacement between the steel bar and grout. The reasons for the differences in maximum displacements of the same batch of specimens may be as follows: (1) the different carbon contents of the steel bars, which led to different elongation rates; (2) the presence of grouting defects and local voids in the samples, which caused slight sliding between the steel bars and the grout; (3) the relative sliding between the steel bars and the tester jig, which introduced certain errors.

The test results of residual deformation and total elongation under maximum tension of the socket joint specimen are shown in Table 9. It can be seen that whether the steel bar pull-off damage or steel bar pull-out damage occurs, the residual deformation is μ_0_ ≤ 0.1 mm, meeting the specification requirements. The total elongation (A_sgt_) under maximum tension is mainly composed of bar deformation and the bond slip of the steel bar. For the steel bar pull-off damage specimen, the steel bar has yielded, so the steel bar deformation constitutes the main part of the total elongation. For the steel bar pull-out damage specimen, the bond slip of the steel bar dominates the total elongation. As can be seen from Table 9, the total elongation under the maximum tensile force shows no obvious pattern. For the specimen of steel bar pull-off damage, its A_sgt_ is greater than 6%, meeting the requirements of the specification JGJ355-2015 [28], which indicates that sleeve joints using the iron tailing sand grout sleeve joints exhibit good ductility. In addition, for the specimens with steel pull-out damage, the deformation properties of some of the specimens meet the specification requirements.

### 4.2. Effect of Rebar Diameter on Unidirectional Tensile Properties of Joints

Different diameters of steel bars correspond to different types of sleeves, and their corresponding minimum anchorage lengths are different. Under the three anchorage lengths of 5 d, 6 d, and 7 d, the mechanical properties of the iron ore mine grout sleeve joints were explored with rebar diameters of 16 mm, 18 mm, 20 mm, 25 mm, and 28 mm as variables. The specific experimental parameters are shown in Table 10.

Table 10 shows the experimental results and key performance indicators. Among them, the maximum elongation of 25 mm and 28 mm specimens was not measured because the corresponding sleeve lengths of 25 mm and 28 mm rebars were too large. This resulted in the maximum elongation being measured over too large a scale distance, and the maximum range between fixtures of the testing machine was limited. Table 11 shows the key performance indicators and test results.

**Figure 12:**
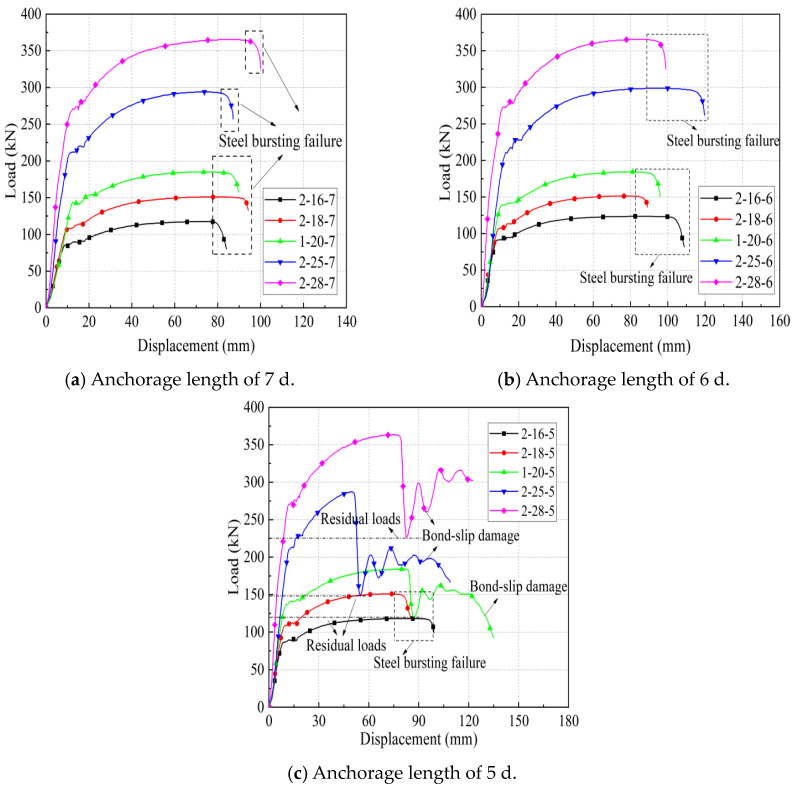
The influence of steel bar diameter on the mechanical properties of joints. Different color curves represent different parameter specimens. See Table 8 for specimen information.

As shown in Figure 12, the influence of steel bar diameter on the mechanical properties of joints is investigated. The ultimate bearing capacity and yield strength of the joint specimens increase with the increase of the steel bar diameter. From specimen 2-16-7 to specimen 2-28-7, as the diameter of the steel bar increases from 16 mm to 28 mm, the yield capacity increases from 83.68 kN to 270.55 kN, and the ultimate capacity increases from 117.7 kN to 366.5 kN. From specimen 2-16-6 to specimen 2-28-6, as the diameter of the steel bar increases from 16 mm to 28 mm, the yield capacity increases from 91.4 kN to 366.5 kN. From Specimen 2-16-5 to Specimen 2-28-5, as the diameter of the spliced steel bar increases from 16 mm to 28 mm, the yield capacity increases from 86.45 kN to 271.23 kN, and the ultimate capacity increases from 118.2 kN to 364.7 kN.

For the joint specimens with rebar diameters of 16 mm and 18 mm, the specimens were damaged by bar pull-out when the anchorage length was 5 d, 6 d, and 7 d. For the joint specimens with rebar diameters of 20 mm, 25 mm, and 28 mm, when the anchorage length was 5 d, the specimens all suffered from bond breakage, and the steel bars were pulled out. This indicates that as the diameter of the steel bar decreases, the stronger the grip of the iron tailing sand grout on the steel bar, the greater the relative bond strength.

It is worth noting that the rebar supporting the sleeve with diameters of 25 mm and 28 mm has significantly greater quality compared to those with smaller diameters. During specimen production, it was more difficult to fix the sleeve of the steel nails, making it harder to support the weight and resulting in a smaller anchorage length, thus introducing a certain degree of error in the results.

### 4.3. Effect of Grout Type on Uniaxial Tensile Properties of Joints

Iron tailing sand grout is the most critical bonding medium for grouting sleeve joints, and its fluidity directly affects whether the grout is dense or not. Its mechanical properties play a decisive role in the quality of joints. Therefore, it is necessary to compare the iron tailing sand grout sleeve joints with the full mechanism of sand grout sleeve joints. In this section, the mechanical properties of sleeve joints under different anchorage lengths are investigated using 20 mm diameter steel bars as the benchmark, with iron tailing sand grout and mechanism sand grout as variables. The specific test parameters are shown in Table 12. The key performance indicators of the test results are shown in Table 13.

**Figure 13:**
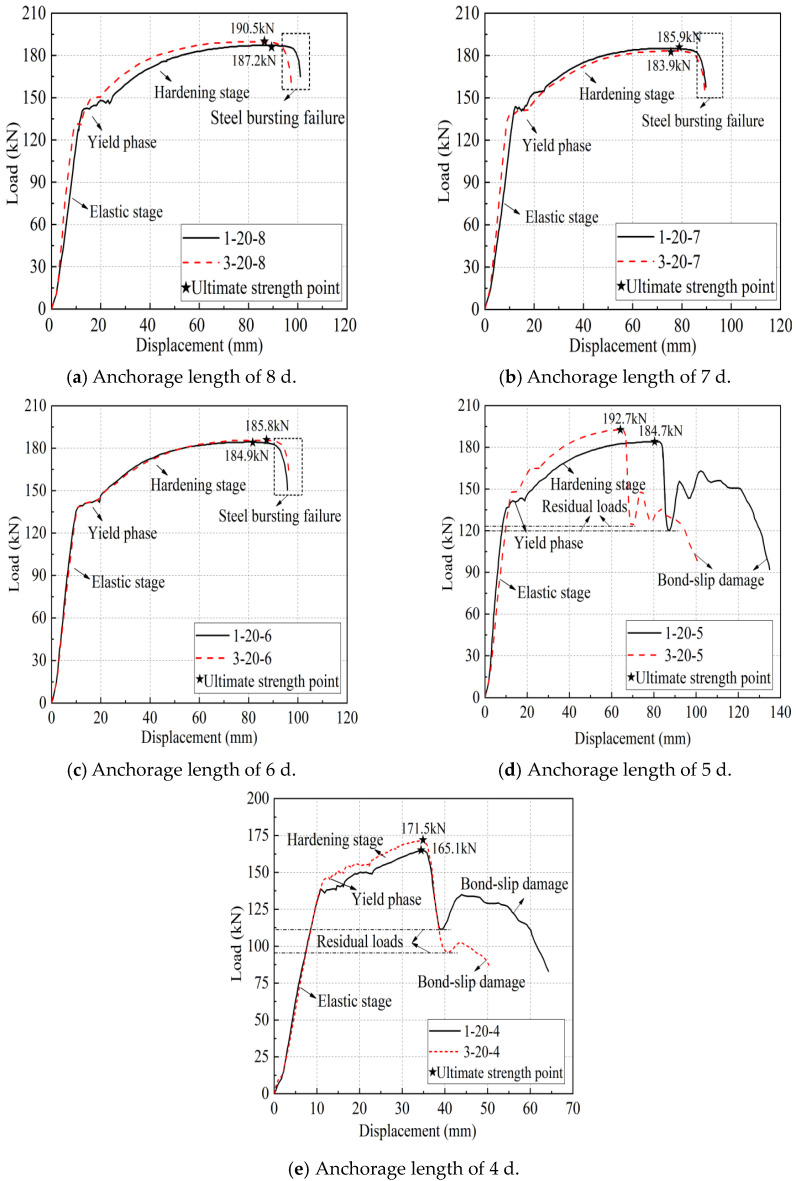
The influence of anchorage length on the mechanical properties of joints. Different color curves represent different parameter specimens. See Table 8 for specimen information.

As shown in Figure 13, the influence of different anchorage lengths on the mechanical properties of joints is analyzed. For anchorage lengths of 6 d, 7 d, and 8 d, pull-off damage occurred in the sleeve joints of both grout types, and for anchorage lengths of 5 d and 4 d, pull-out damage occurred in the sleeve joints of both grout types. At an anchorage length of 5 d, the iron tailing sand grout joints entered the yield stage earlier, and the elongation of the reinforced section was larger, indicating that the iron tailing sand grout joints showed better ductility after entering the plastic stage. The ultimate tensile strengths of the mechanism sand grout joints were slightly greater than those of the iron tailing sand grout joints when the anchorage lengths were 5 d and 4 d. However, when the anchorage length was 4 d, the residual bond strength of the iron tailing sand grout joint was significantly greater than that of benchmark manufactured sand grout. In summary, it can be concluded that the performance of iron tailing sand grout sleeve joints is similar to that of mechanism sand grout sleeve joints, indicating that it is feasible to use 40% iron tailing sand instead of mechanism sand to prepare the new iron tailing sand grout in the uniaxial tensile test.

### 4.4. Repeated Tensile Tests with High Stresses and Large Deformations

When the grout composition is changed, the sleeve joints also need to be tested for performance under high-stress repeated tensile and large-deformation repeated tensile loading. Table 14 shows the parameters of the two repeated tensile tests.

**Figure 14:**
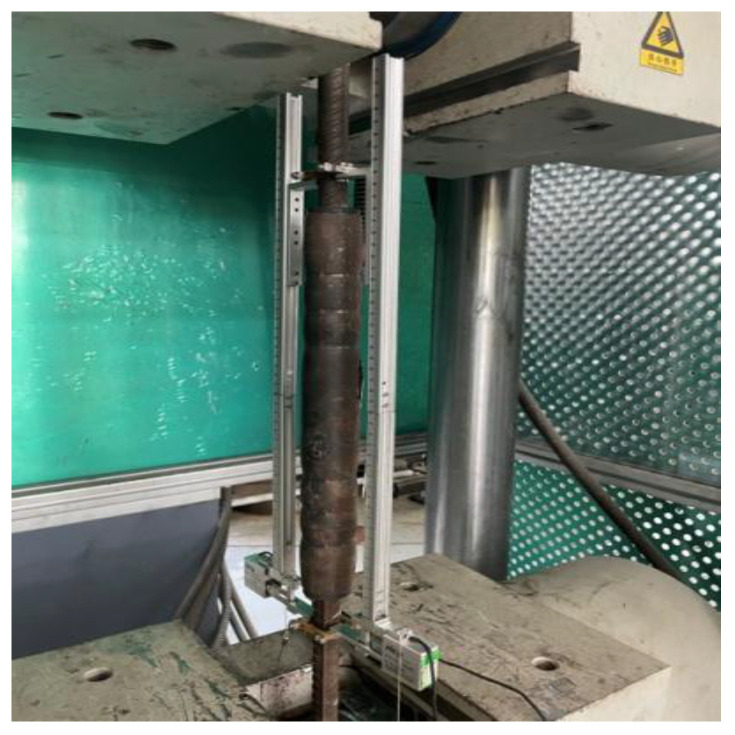
Measurement of residual deformation by an extensometer.

The residual deformation after eight cycles of high-stress loading and four or eight cycles of large-deformation loading is an important index to verify the deformation performance of the sleeve joint. As shown in Figure 14, the residual deformation was measured using an extensometer connected to the universal testing machine(China Guangdong Province Zhuhai City Sansi Taijie Electrical Equipment Co., Zhuhai, China).

For the high-stress and large-deformation repeated tensile tests, the main purpose is to verify the deformation performance. Table 15 shows the results of repeated tensile tests and key performance indicators. As regards residual deformation, for high-stress repeated tensile test, *μ*_20_ = 0.230 ≤ 0.3; for large-deformation repeated tensile test, *μ*_4_ = 0.046 ≤ 0.3, and *μ*_8_ = 0.109 ≤ 0.6. Both deformations meet the specification requirements. The maximum elongation was 6.29% for the large-deformation repeated tension specimen and 8.29% for the high-stress repeated tension specimen. The joints of all specimens exceed 6%, meeting the specification requirements.

For the strength requirements of repeated tensile test, after cyclic loading and subsequent uniaxial tensile testing, both specimens failed by rebar pull-off, which indicates that the repeated tensile test of sleeve joints using iron tailing sand grout meets the strength requirements.

**Figure 15:**
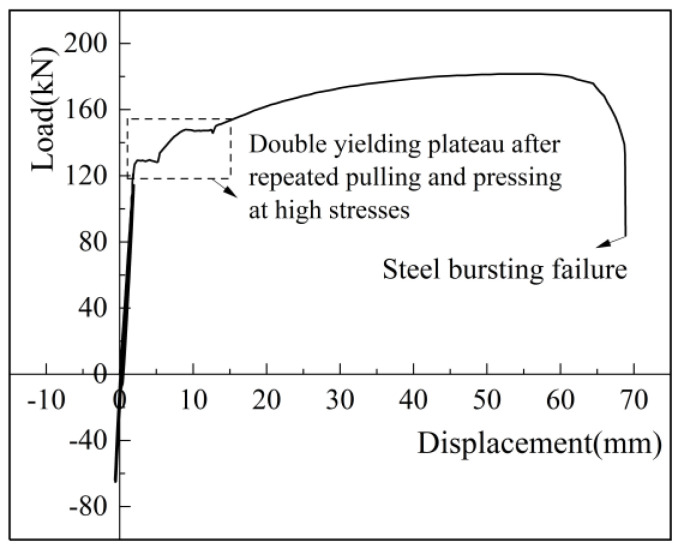
Load-displacement curves for repeated high-stress tensile tests.

**Figure 16:**
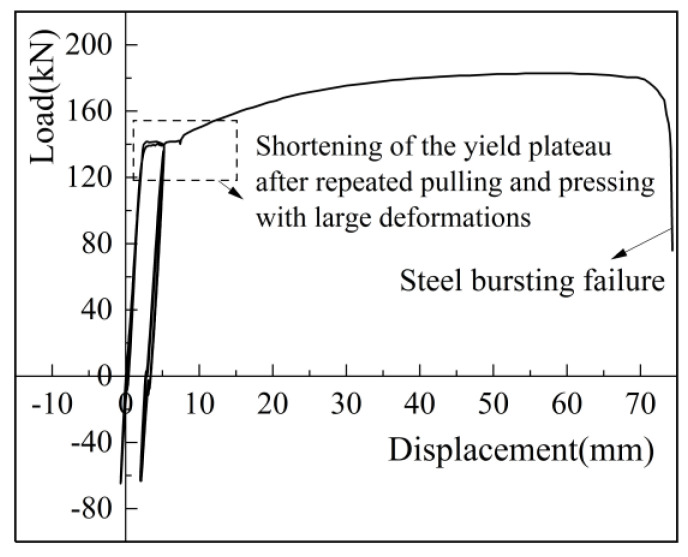
Load-displacement curves for repeated tensile tests with large deformations.

The load-displacement curves under the high-stress repeated tensile and large-deformation repeated tensile and compressive loading regimes for iron tailing sand grouted sleeve joints are shown in Figure 15 and Figure 16. The specimens were loaded positively in tension and negatively in compression. The residual deformation increased gradually with the increase in the number of cycles. The axial displacement of the iron tailing sand grout sleeve joint changed roughly linearly with the load, and the stiffness of the joint did not degrade significantly, indicating that the bond slip between the iron tailing sand grout and the anchoring steel bar was very small during the cyclic loading process, and the deformation of the joint was mainly the elastic deformation of the steel bar. During repeated tensile cyclic loading, no obvious splitting cracks were observed at the end of the anchoring steel bar.

After cyclic loading for uniaxial tensile, the iron tailing sand grout sleeve joints ultimately failed due to rebar pull-out. Because the rebar was close to yielding in the process of repeated tensioning, the yield platform changed somewhat in the load-displacement curve of the joint specimen after cyclic loading. Specifically, after the high-stress repeated tensile cyclic loading, a double yield plateau appeared; after the large-deformation repeated tensile cyclic loading, the yield plateau became shorter.

In addition, the shape of the curve remained similar to the load-displacement curve of the iron tailing sand grout sleeve joint under uniaxial tensile loading. It is worth noting that the displacement in the elastic phase of this test was smaller than that measured by the ordinary 200 kN electro-hydraulic servo universal testing machine, as the microcomputer-controlled electro-hydraulic servo universal testing machine was connected to an extensometer, providing more accurate data than a displacement meter.

**Figure 17:**
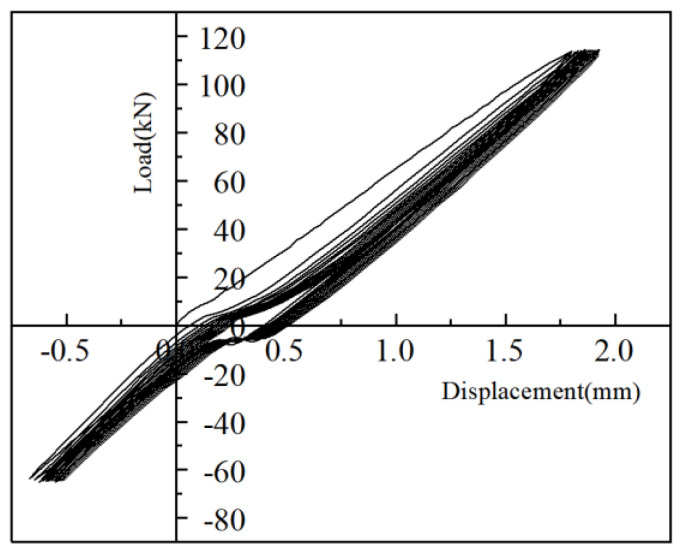
Load-displacement cyclic curves for high stresses.

**Figure 18:**
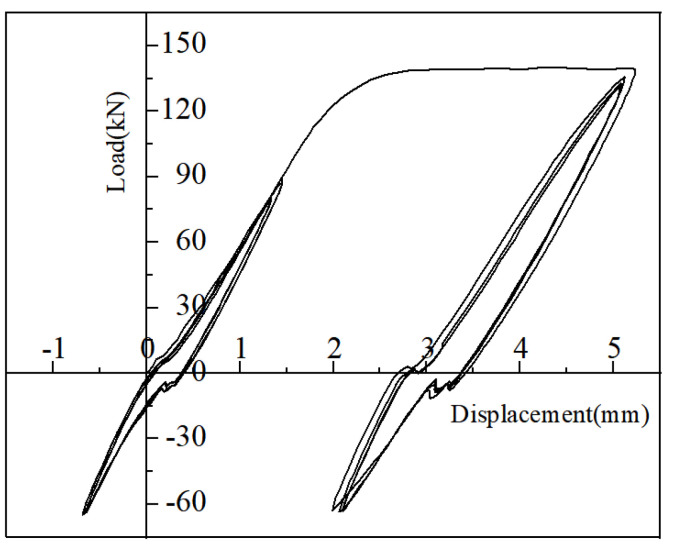
Load-displacement cyclic curves for large deformations.

In order to show more clearly the load-displacement changes in the joint under repeated tensile and compressive loads, the change curves of the cyclic process are plotted separately in Figure 17 and Figure 18.

The above results show that the strength and deformation properties of the grouted sleeve joints with iron tailing sand grout performed well in repeated tensile tests, proving the technical feasibility of using iron tailing sand grout as sleeve grout.

### 4.5. Effect of Grout Age and Rebar Offset on Uniaxial Tensile Properties of Joints

The mechanical properties of the grouting sleeve connection are affected by factors such as rebar, sleeve, and grout. At the same time, in the actual construction process, issues such as construction personnel’s proficiency, construction quality, and others can easily cause the misalignment of the steel bar with the sleeve center, forming eccentric steel bar sleeve joints. Additionally, an insufficient grout maintenance cycle can also impact the performance of the joint. In this section, we take 20 mm bar diameter, 8 d anchorage length, and iron tailing sand grout as the benchmark, and consider the ages of 14 d and 28 d as the parameters to examine the effect of the age of iron tailing sand grout on the joint stressing performance. We also take bar alignment and bar eccentricity as variables to examine the effect of bar eccentricity on joint stressing performance. The specific test parameter settings are shown in Table 16. Table 17 shows the test results and key performance indicators.

**Figure 19:**
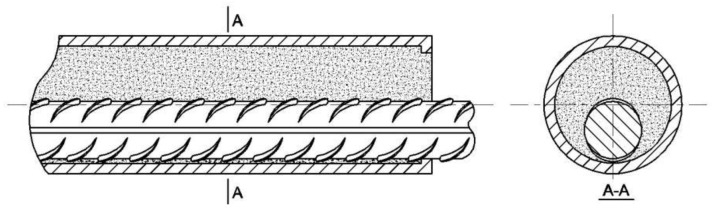
Schematic diagram of uniaxial tension offset rebar joints.A-A (profile).

For sleeve joint specimens with centered rebar, the steel bar at both ends was inserted into the center of the grouting sleeve. For sleeve joint specimens with eccentric rebar, the steel bar was centered at one end, and the center of the transverse rib of the offset steel bar contacted the sleeve wall at the other end, as shown in Figure 19. In this case, the thickness of the protective layer in the direction of eccentricity was 0.

**Figure 20:**
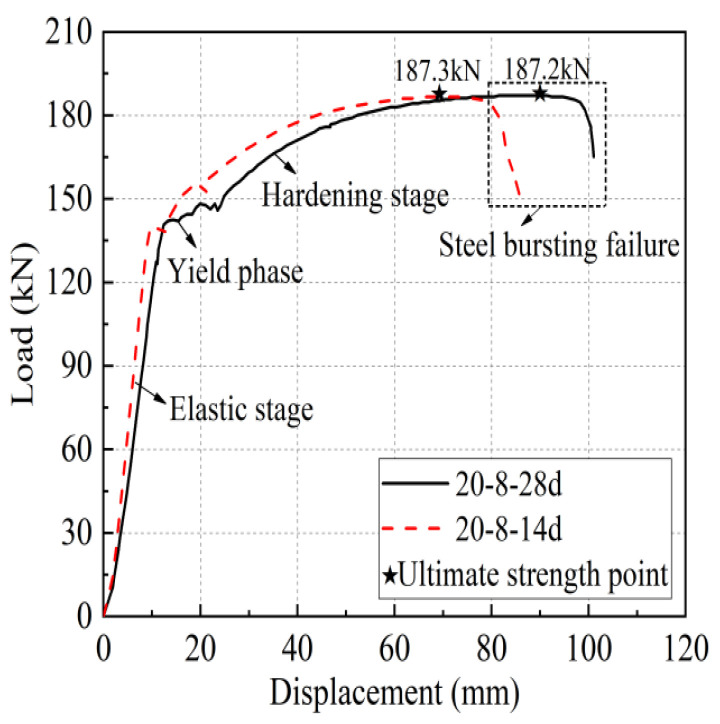
Load-displacement curves of grout sleeve joints at different ages.

The curves of 14 d and 28 d iron tailing sand grout joints of different ages were analyzed together to understand the influence of different ages on the mechanical properties of the joints, and their load-displacement curves are shown in Figure 20. The 14 d steel joints and 28 d steel joints were compared and analyzed. The 14 d steel joints entered the yielding stage and strengthening stage earlier, the yield strength and ultimate load of both were almost the same, and the load-displacement curves were similar. Additionally, as discussed in Section 2, the 14 d iron tailing sand grout reached the strength requirement of 85 MPa by 28 d, indicating that iron tailing sand grout sleeve joints at 14 d already demonstrated adequate performance.

**Figure 21:**
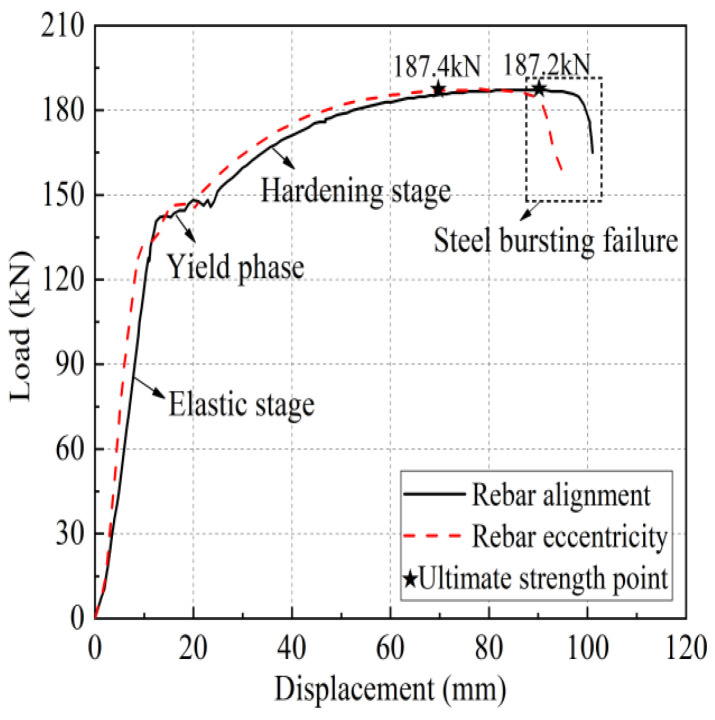
Load-displacement curves for offset rebar sleeve joints.

The influence of protective layer thickness on the mechanical properties of the joint was analyzed, and its load-displacement curve is shown in Figure 21. The sleeve joints with rebar eccentricity were compared and analyzed with the sleeve joints with centered rebar. The offset rebar joints entered the yielding stage and strengthening stage earlier, and the yield strength was significantly lower than that of the centered rebar joints. The ultimate loads of 187.2 kN and 187.4 kN for the centered rebar joints and the offset rebar joints were almost identical. This indicates that whether the steel bar is eccentric or not (i.e., the change in the protective layer thickness) has almost no effect on the load-carrying capacity of the specimens, and this conclusion aligns with that of Zheng et al. [30].

## 5. Analysis of Internal Forces in Sleeve Joints

### 5.1. Pilot Programme Design

A total of five groups were established, each containing one joint specimen for the uniaxial tensile test, resulting in five specimens overall. The 20 mm bar diameter and iron tailing sand grout were used as the reference, with different anchorage lengths as parameters. The specific parameter settings and specimen numbers are shown in Table 18.

**Figure 22:**
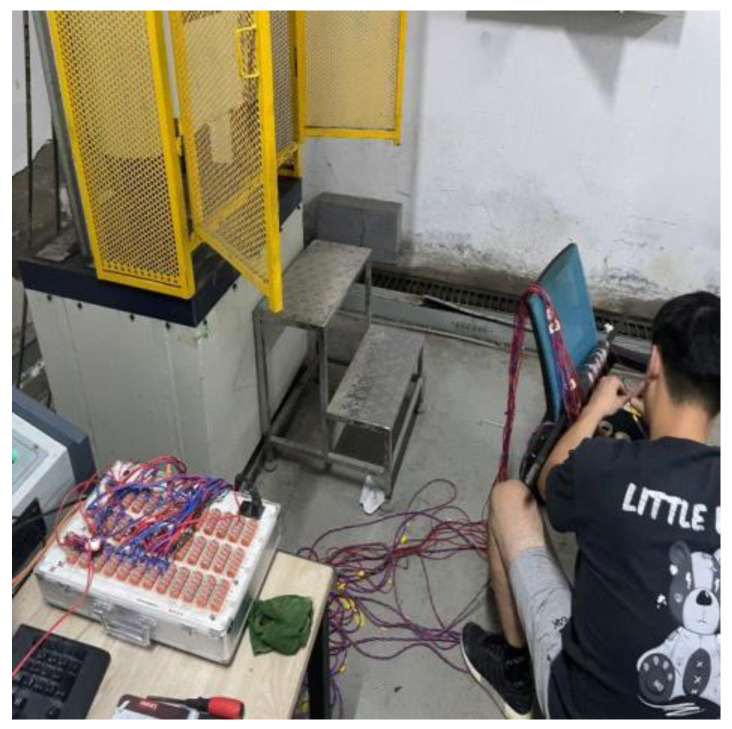
Connecting the data collector.

**Figure 23:**
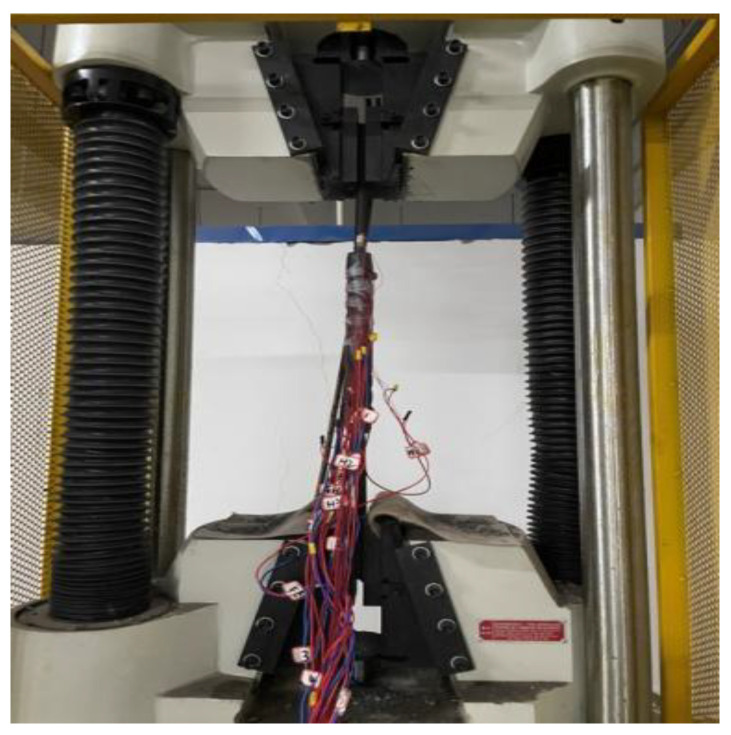
Uniaxial tensile.

Unlike the loading method in Section 4, only unidirectional loading was used throughout, as residual deformation did not need to be measured for this test. To minimize strain gauge damage to the anchorage, smaller 120-3AA type strain gauges were used. The strain gauge data were collected through a data acquisition instrument connected to a computer, as shown in Figure 22. Unidirectional pull-out loading was applied using a universal testing machine, as shown in Figure 23.

### 5.2. Test Results and Analyses

#### 5.2.1. Experimental Results

**Figure 24:**
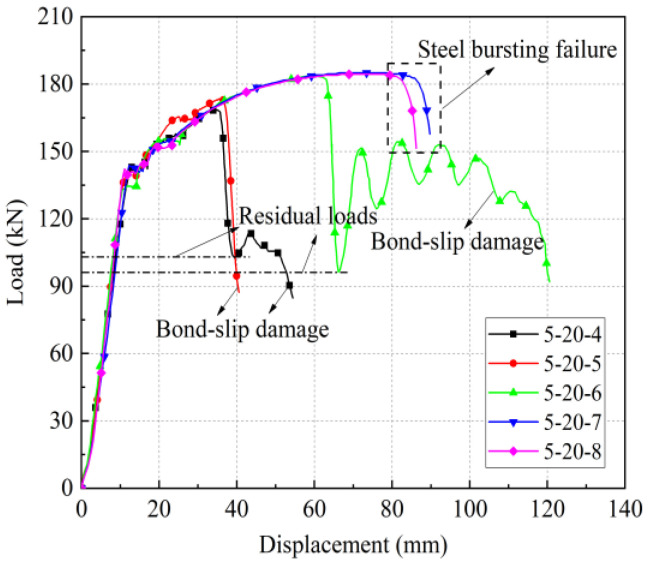
Load-displacement curve for pasted strain gauge set.

Table 19 shows the uniaxial tensile test results and key performance indicators for the affixed strain gauge group. The elongation at maximum force, A_sgt_, was not measured due to the strain gauges attached to the steel bars in the non-anchored section. Steel bar pull-out damage occurred in the specimens with anchorage lengths of 8 d and 7 d, and steel bar pull-out damage occurred in the specimens with anchorage lengths of 6 d, 5 d, and 4 d. Figure 24 shows the load-displacement curves for each set.

**Figure 25:**
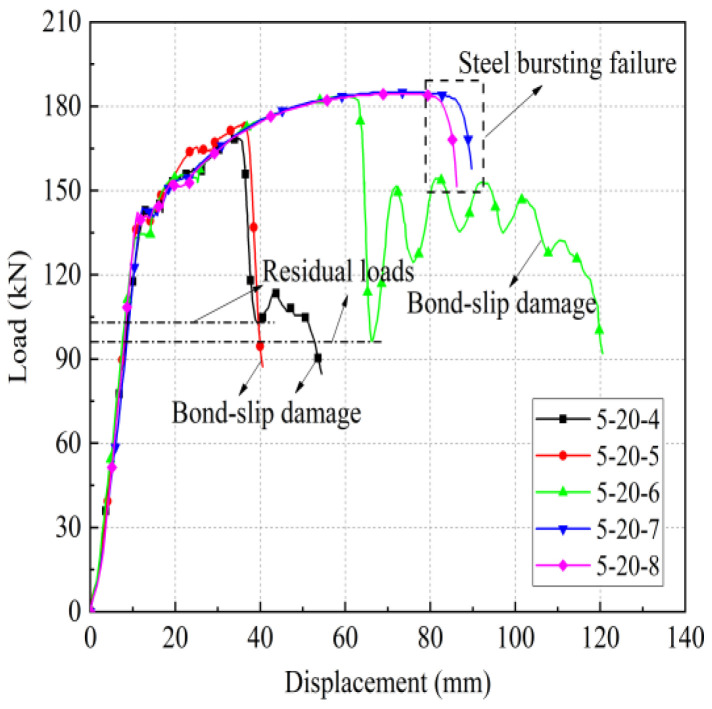
Comparative analysis of strain gauges applied at 6 d anchorage length.

It is worth noting that in Section 2, with a 20 mm steel bar and 6 d anchorage length, the sleeve joints underwent rebar pull-out damage, while in the case of sleeve joints with the affixed strain gauges, rebar pull-out damage also occurred. This indicates that the adhesion of strain gauges weakened the bond strength to some extent, as shown in Figure 25. The curves of the joint specimens with pasted strain gauges showed a double yield plateau, which indicates that the paste of strain gauges caused some damage to the bond between the steel bar and grout.

#### 5.2.2. Sleeve Surface Load Stress Analysis

**Figure 26:**
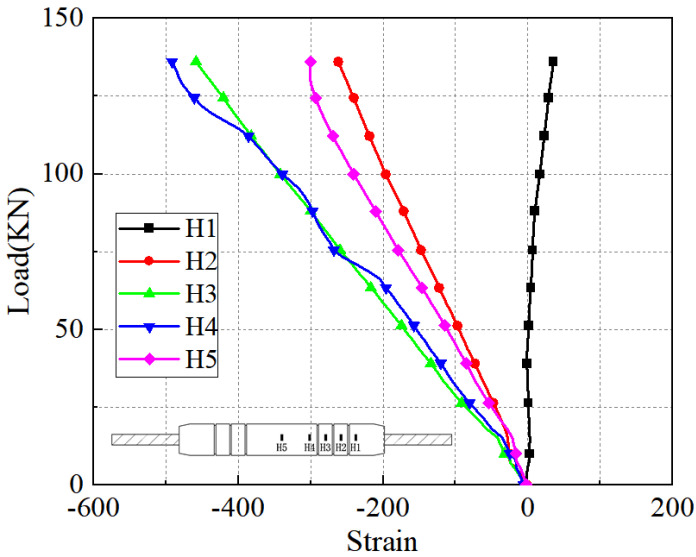
Transverse stress on the sleeve surface at 8 d anchorage length.

**Figure 27:**
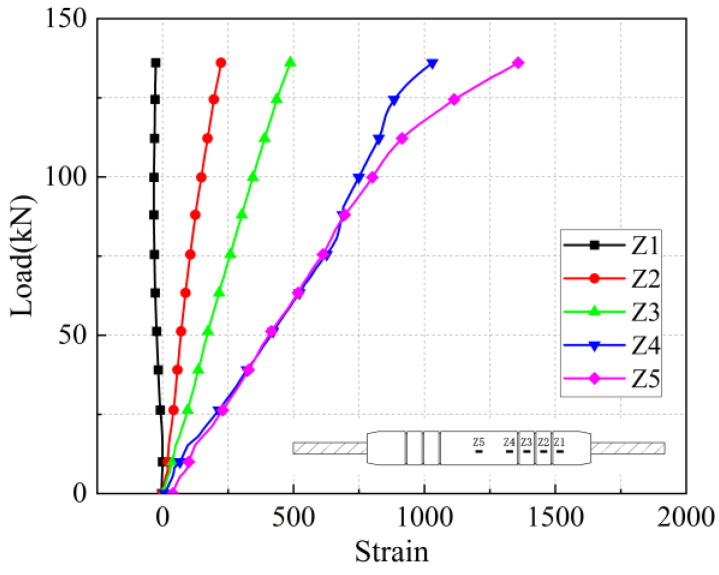
Axial stress on the sleeve surface at 8 d anchorage length.

**Figure 28:**
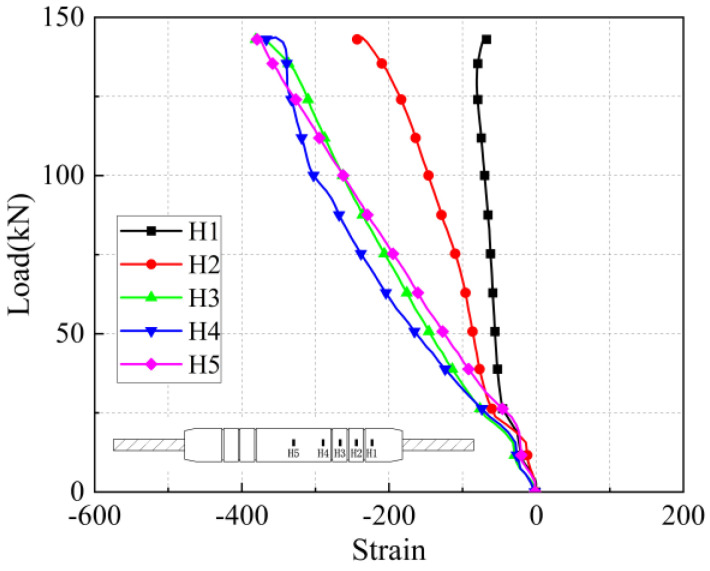
Transverse stress on the sleeve surface at 7 d anchorage length.

**Figure 29:**
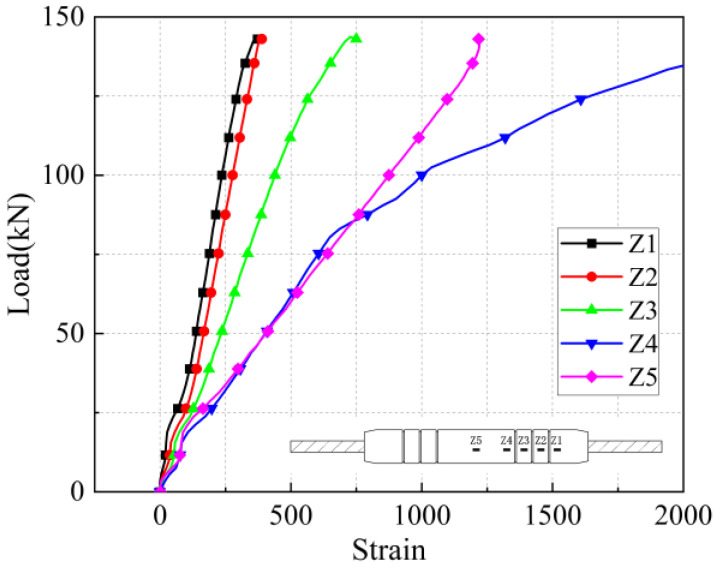
Axial stress on the sleeve surface at 7 d anchorage length.

**Figure 30:**
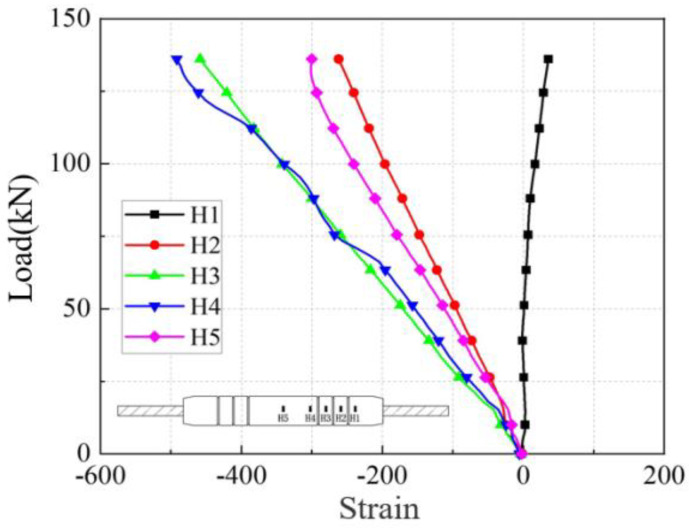
Transverse stress on the sleeve surface at 6 d anchorage length.

**Figure 31:**
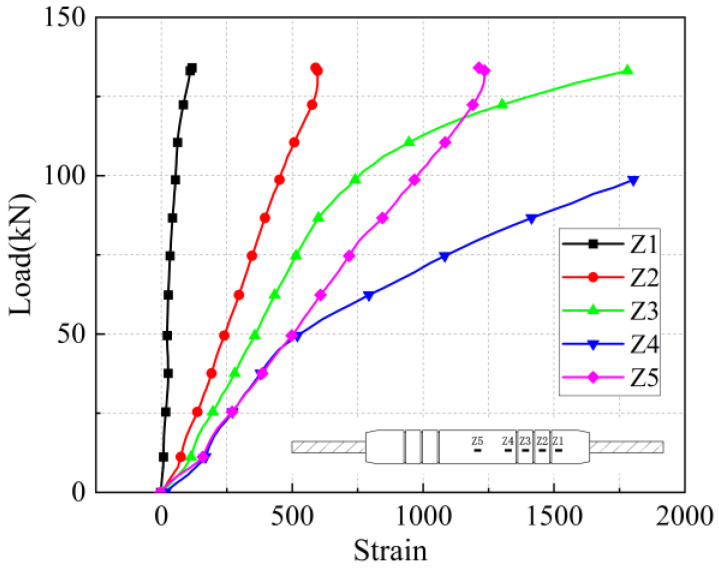
Axial stress on the sleeve surface at 6 d anchorage length.

**Figure 32:**
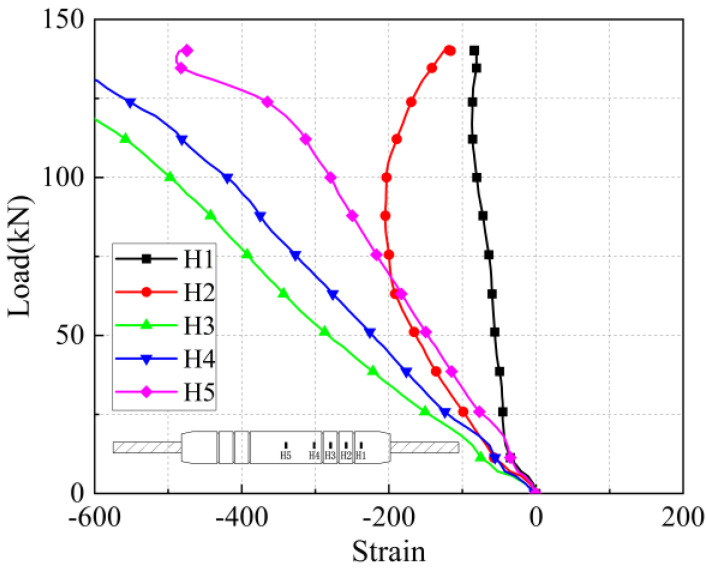
Transverse stress on the sleeve surface at 5 d anchorage length.

**Figure 33:**
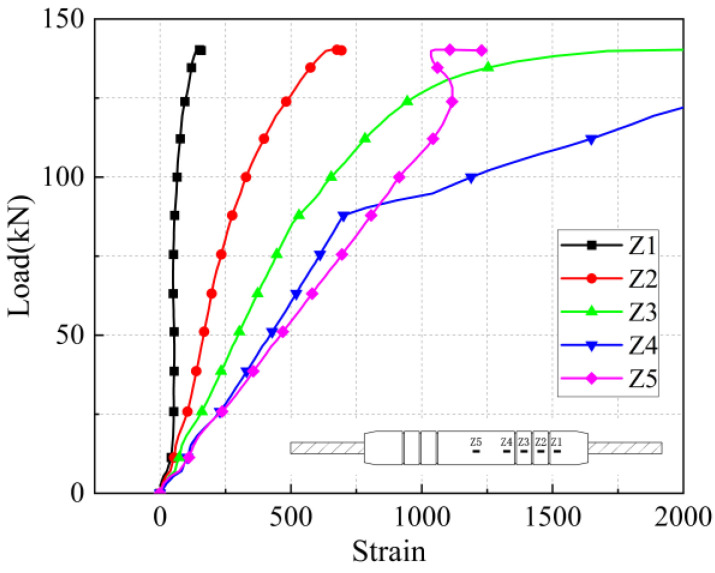
Axial stress on the sleeve surface at 5 d anchorage length.

**Figure 34:**
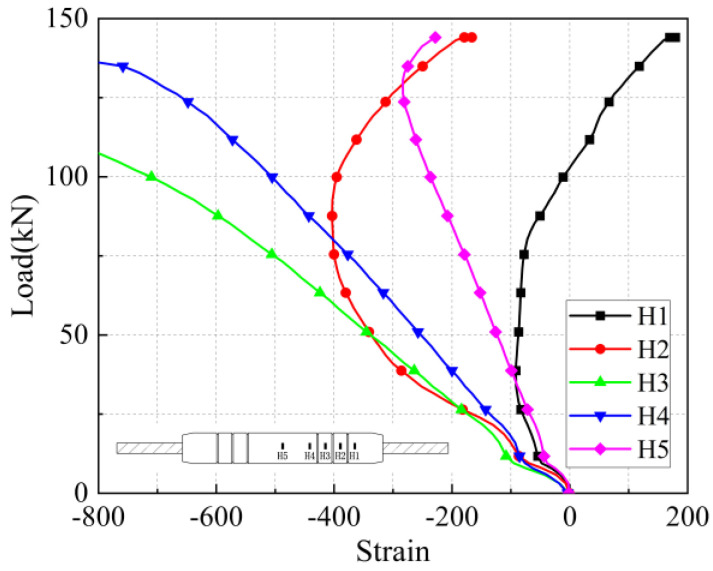
Transverse stress on the sleeve surface at 54 d anchorage length.

**Figure 35:**
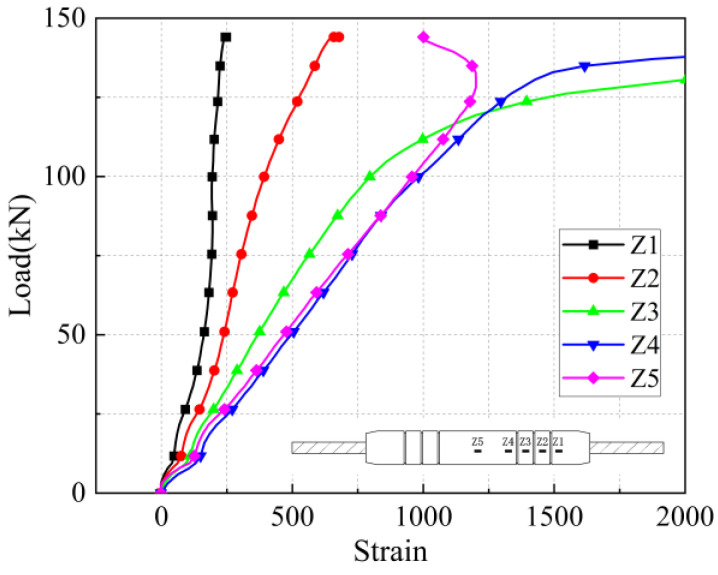
Axial stress on the sleeve surface at 54 d anchorage length.

The load-strain curves in the axial and transverse directions on the sleeve surface for the five anchorage lengths are shown in Figure 26, Figure 27, Figure 28, Figure 29, Figure 30, Figure 31, Figure 32, Figure 33, Figure 34 and Figure 35. The transverse strain on the sleeve surface as a function of the pull-out load shows the stress spread over the sleeve surface. The transverse strain in the smooth middle part of the sleeve and in the deformed part near the ring rib is negative, i.e., compressive strain. The strain at H1, which is nearest to the outlet, shows positive, i.e., tensile, but is almost zero, indicating a small strain.

Similar to the transverse strain, the axial strain on the surface of the sleeve, closest to the outlet Z1, is also almost zero, indicating that the strain is also very small. The strain is positive in the remaining parts and is greater in the center of the sleeve at Z5 and nearest to it at Z4.

In the unribbed section, the bond stress between the iron tailing sand grout and the sleeve is mainly due to friction, and in the deformed section, the bond stress between the two is increased by the mechanical occlusion force. As the joint is subjected to tensile loading, the grout will slide with the stretching of the steel bar, thus triggering the interaction between the sleeve and the iron tailing sand grout. In the deformation section, the interaction consists of frictional action and contact pressures acting on the concentric ribs, and these concentrated pressures put the sleeve section between the ribs in a state of localized radial bending, and, therefore, transverse compressive strains are detected on the outer surface of the sleeve. This phenomenon is similar to the test results of Zheng et al. [31]. Since the inner cavity structure of the unused sleeve is different, the strain distribution on the sleeve surface is also slightly different from the findings of some scholars.

#### 5.2.3. Stress-Strain Analysis of Reinforcing Bar Surfaces

**Figure 36:**
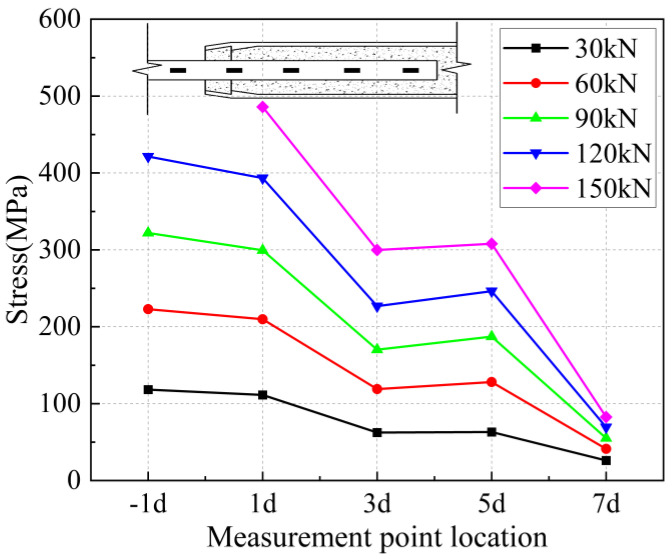
Rebar stress at 8 d anchorage length.

**Figure 37:**
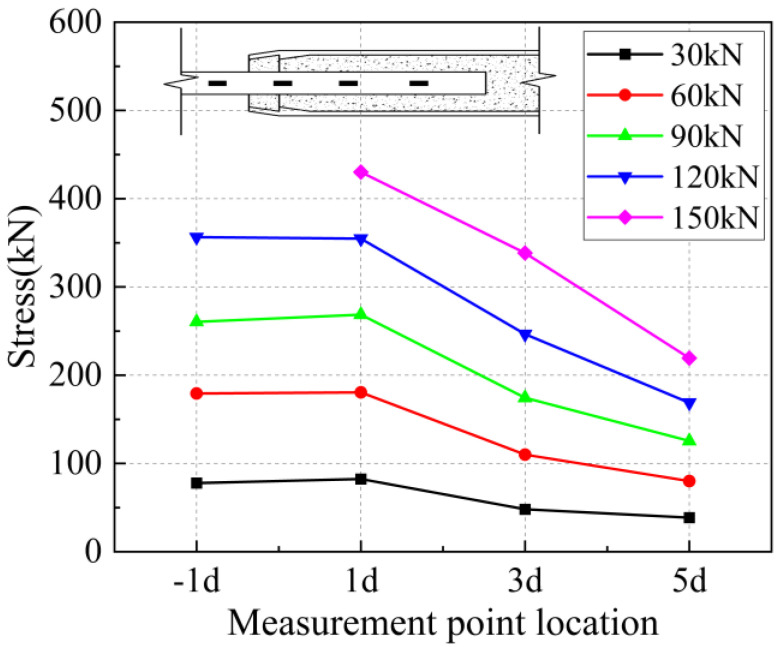
Rebar stress at 7 d anchorage length.

**Figure 38:**
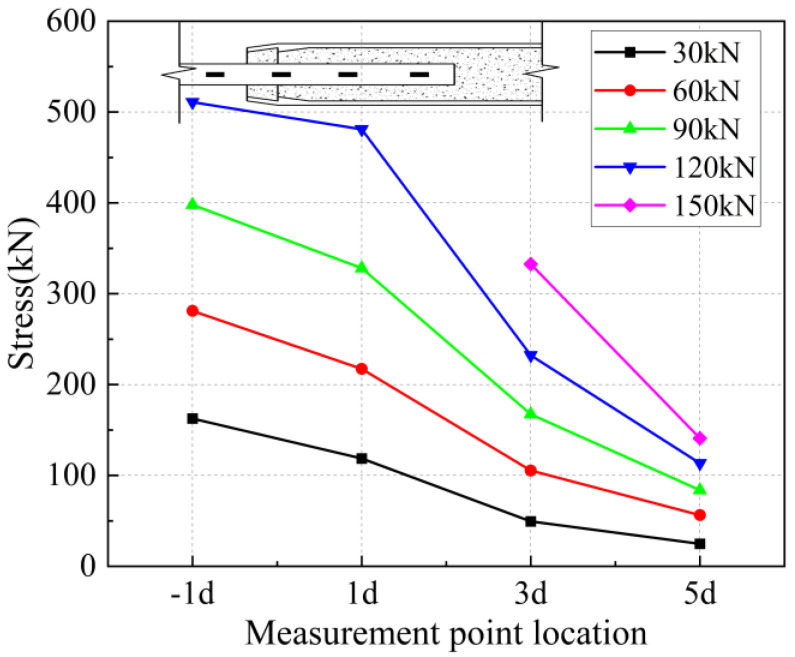
Rebar stress at 6 d anchorage length.

**Figure 39:**
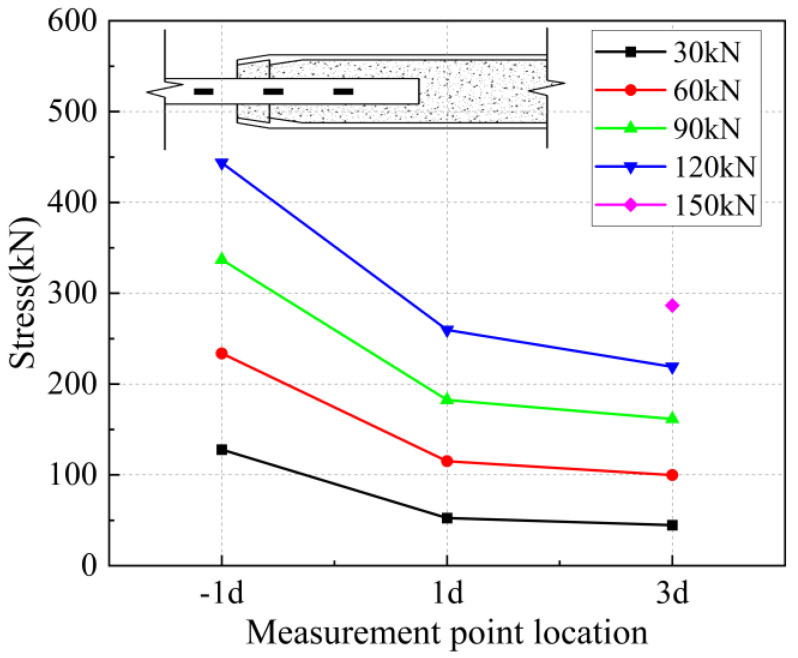
Rebar stress at 5 d anchorage length.

**Figure 40:**
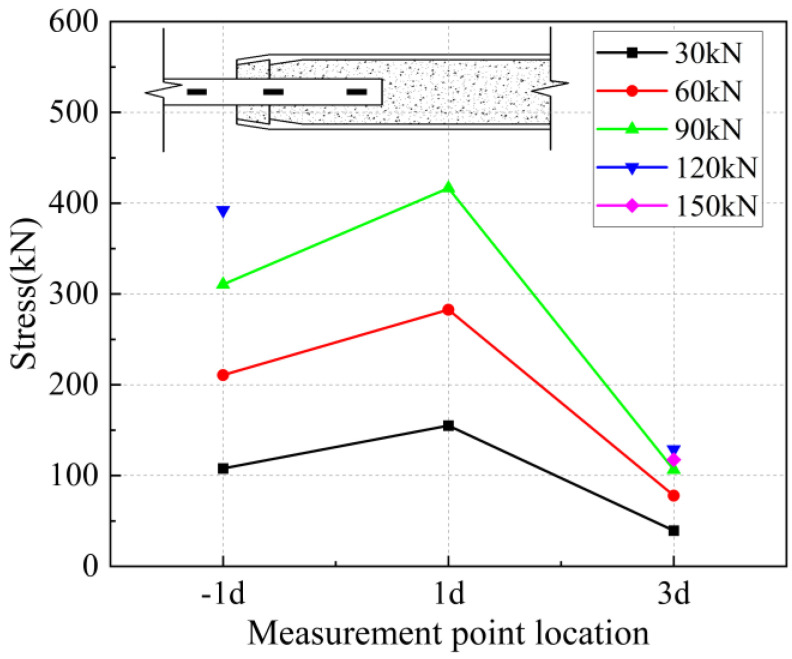
Rebar stress at 4 d anchorage length.

The stress-strain curves at the location of the rebar measurement point under five anchorage lengths are shown in Figure 36, Figure 37, Figure 38, Figure 39 and Figure 40. Due to the limited range of strain gauges, combined with the load-displacement curves, it was found that the strain of the steel bar began to increase sharply or even fail when the joint of the iron tailing sand grout sleeve reached the yield state, at which time the measurement results fluctuated greatly. Therefore, the data with appropriate strain size should be selected as the basis for calculating the bond stress to ensure the authenticity of the results.

The strain values on the surface of the anchored steel bar were calculated and processed to derive the corresponding steel bar stresses. The strain value ε is determined to be located at the position of the arranged strain gauges corresponding to each loading stage of 30 kN, 60 kN, 90 kN, 1200 kN, and 150 kN, and the stress of the anchored steel bar is Eε, where E is the elasticity modulus of the connecting steel bar of 200 GPa. In addition, “−1 d” in the figure represents the outer reinforcement at 1 d from the anchorage section.

From Figure 31 and Figure 32, it can be seen that for specimen 5-20-8 and specimen 5-20-7, the stress of the external steel bar and the stress of the steel bar in the anchored steel bar at a distance of 1 d from the external steel bar were roughly equal, indicating that the anchoring effect of the anchoring section at a distance of 1 d from the external position of the iron tailing sand grout was not fully utilized. Beyond the 1 d position, the surface stress of the steel bar began to gradually decline, indicating that the iron tailing sand grout at the end of the sleeve constraint anchorage effect was small, and beyond the 1 d distance, the anchorage effect became more pronounced.

It is worth noting that, from Figure 31, it can be observed that the measured value at 5 d exhibits a sudden change; this is due to the 5 d position at the strain gauges happened to be located near the third ring rib, under which the two distances from the port are 100 mm. Due to the existence of shear ring ribs, the iron tailing sand grout sleeve joints also exhibit depressions at the ring rib, where irregular mechanical occlusion forces, interface bonding forces, and other joint effects influence the surface stress of the rebar.

From Figure 33, Figure 34 and Figure 35, it can be seen that for specimen 5-20-6 and specimen 5-20-5, where bond-slip damage occurred, the stresses showed a uniformly decreasing trend from −1 d. For specimen 5-20-4, there is an obvious extreme value at −1 d. It is suggested that this may be due to issues in the arrangement of the strain gauges, resulting in abnormal final data, but it does not affect the overall performance of the sleeve joint. It can be seen that the specimens generally show good uniformity, good overall performance of iron tailing sand grout, and relatively high utilization of iron tailing sand grout in the initial anchorage section.

## 6. Conclusions

Based on the existing research results, this paper examines the use of iron tailing sand grout with an optimal substitution rate of 40%, comparing it with the benchmark manufactured sand grout used in grout sleeves. The mechanical properties of the iron tailing sand grout sleeve joints were tested under three loading modes: uniaxial tensile, repeated pulling and pressing with large deformation, and repeated high-stress pulling and pressing. This study analyzes the influence of different factors on the performance of sleeve joints and verifies the feasibility of using iron tailing sand grouting rebar sleeve joints. In addition, by pasting strain gauges on the surface of the sleeve and the anchoring steel bar, the interfacial bond stresses between the sleeve surface, grout, and steel bar were investigated, along with their distribution patterns. The study demonstrates that the mechanical properties of sleeve joints with new iron tailing sand grout meet the specification requirements, and the feasibility of using iron tailing sand grout is confirmed. The specific conclusions are as follows:

(1) As the diameter of the steel bar decreases, the grip of the iron tailing sand grout on the internal steel bar gradually increases, and the bond strength increases. With larger diameters, grout sleeve joints require longer anchorage lengths. For steel bar diameters of 20 mm, 25 mm, and 28 mm, the minimum anchorage length should not be less than 6 d.
(2) Both large-deformation and high-stress cyclic loadings followed by uniaxial tensile test resulted in steel bar pull-off damage, indicating that the use of iron tailing sand grout socket joints can withstand repeated tensile tests and meets the strength requirements. Meanwhile, the residual deformation μ_4_, μ_8_, and μ_20_ after 4, 8, and 20 cycles of tensile compression of iron tailing grout sleeve joints were 0.046, 0.109, and 0.230, respectively, and the total elongation under the maximum tensile force, A_sgt_, was 6.29% and 8.29%, respectively, indicating that the deformation performance also meets the specification requirements.
(3) Iron tailing sand grout sleeve joints demonstrate adequate performance after being maintained for 14 d. In terms of tensile strength, the performance of the steel bar pull-off damage was observed; in terms of deformation performance, the residual deformation is 0.06, and the total elongation under the maximum tensile force is 7.3%. Both strength and deformation performance meet the specification requirements. Compared with the 28 d age, the 14 d age sleeve joint entered the yielding stage and strengthening stage earlier, but the yield strength and ultimate load were almost the same in both cases.
(4) The effect of rebar eccentricity error on the load-carrying capacity of the sleeve joint is small. The ultimate loads of the sleeve joint with rebar eccentricity at the loading end (the thickness of the protective layer is 0) and the sleeve joint with centered rebar were almost the same, which were 187.2 kN and 187.4 kN, respectively. However, the sleeve joint with rebar eccentricity entered into the yielding stage and the strengthening stage earlier, and the yield strength was significantly lower than that of the sleeve joint with centered rebar.
(5) The restraining anchoring effect of iron tailing sand grout at the end is small, becoming effective only after more than 1 d of distance. The utilization rate of iron tailing sand grout in the initial anchoring section is relatively high, and its overall performance is better.

This paper conducts comprehensive experimental studies on the mechanical properties of iron tailing slag cement-based grout and coupler, proving the feasibility of using iron tailing slag in cement-based grout. It provides valuable systematic experience and reference for this field. However, due to limitations such as the experimental environment, this study does not cover all aspects, including the following: Fine-grained iron tailing slag with a specific gravity modulus of 1.1 was selected in this study, but the performance of cement-based grout is affected by different sizes and surface roughness of aggregate. The basic mechanical properties of grout made with iron tailing slag of different sizes need to be further studied. The effects of other grade and diameter steel bars also require further study. Meanwhile, further research is needed on the torsional characterization of the samples.

## Figures and Tables

**Table 1:** Tensile strength of joints.

Grade of Joints	Level I	Level II	Level III
Tensile strength	*f*^0^*_mst_* ≥ *f_stk_* Steel bar breakage or *f*^0^*_mst_* ≥ 1.10*f_stk_* Joint breakage	*f*^0^*_mst_* ≥ *f_stk_*	*f*^0^*_mst_* ≥ 1.25*f_yk_*

Note: *f*^0^*_mst_* is the measured ultimate tensile strength of the joint specimen; *f_stk_* is the standard value of tensile strength of steel bars; *f_yk_* is the standard value of yield strength of steel bars in the joint specimen.

**Table 2:** Deformation properties of joints.

Grade of Joints		Level I	Level II	Level III
Uniaxial tensile	Residual deformation(mm)	*μ*_0_ ≤ 0.10 (d ≤ 32)	*μ*_0_ ≤ 0.14 (d ≤ 32)	*μ*_0_ ≤ 0.14 (d ≤ 32)
		*μ*_0_ ≤ 0.14 (d > 32)	*μ*_0_ ≤ 0.16 (d > 32)	*μ*_0_ ≤ 0.16 (d > 32)
	Total elongation at maximum force(%)	*A_sgt_* ≥ 6.0	*A_sgt_* ≥ 6.0	*A_sgt_* ≥ 3.0
Repeated tension and compression under high stress	Residual deformation(mm)	*μ*_20_ ≤ 0.3	*μ*_20_ ≤ 0.3	*μ*_20_ ≤ 0.3
Large-deformation reversed tension and compression		*μ*_4_ ≤ 0.3, *μ*_8_ ≤ 0.6	*μ*_4_ ≤ 0.3, *μ*_8_ ≤ 0.6	*μ*_4_ ≤ 0.6

Note: *μ*_0_ is the residual deformation of the joint specimen loaded to 0.6*f_yk_* and unloaded in the specified distance; *μ*_4_ is the residual deformation of the joint specimen after 4 cycles of repeated pulling and pressing with large deformation; *μ*_8_ is the residual deformation of the joint specimen after 4 cycles of repeated pulling and pressing with large deformation; *μ*_20_ is the residual deformation of the joint specimen after 20 cycles of repeated tensioning under high stress; *A_sgt_* is the total elongation of the joint specimen under maximum force.

**Table 3:** Performance of grouting material.

Grouting Material Type	Fluidity (mm)		Compressive Strength (MPa)			Vertical Expansion Rate (%)	
	0 min	30 min	1 d	3 d	28 d	3 h	24 h
Manufactured sand grout	330	289	43.6	68.0	93.9	0.002	0.051
Iron tailing sand grout	313	278	43.3	68.9	94.2	0	0.049

**Table 4:** Sleeve size.

Model Number	*D* (mm)	*L* (mm)	*d* (mm)	*L*1 (mm)	*L*2 (mm)	*L*3 (mm)
GTQ4J16	42	315	29.5	158	32	30
GTQ4J18	45	345	32.5	174	32	30
GTQ4J20	45	375	32.5	189	40	30
GTQ4J25	58	455	42.5	229	45	30
GTQ4J28	63	505	46.5	255	45	30

**Table 5:** Performance of reinforcement.

Bar Diameter (mm)	Yield Strength (MPa)	Yield Load (kN)	Tensile Strength (MPa)	Ultimate Load (kN)
16	411	82.6	584	117.4
18	437	111.3	594	151.3
20	471	148.0	618	194.1
25	450	220.9	602	295.4
28	445	274.2	594	365.9

**Table 6:** Design of test parameters for sleeve joints.

Grouting Material Type	Anchor Length	Rebar Diameter	Test Loading System	Grout Age	Eccentricity
Manufactured sand grout Iron tailing sand grout	4 d, 5 d, 6 d, 7 d, 8 d	16 mm, 18 mm, 20 mm, 25 mm, 28 mm	Uniaxial tensile, Repeated tension and compression under high stress, Large-deformation reversed tension and compression	14 d, 28 d	middle, biased

**Table 7:** Pilot program design.

Group Number	Rebar Diameter	Anchor Length (mm)	Number of Specimens	Types of Grout	Note
1	20 mm	4 d, 5 d, 6 d, 7 d, 8 d	15	Iron tailing sand grout	Uniaxial tensile
2	16 mm	5 d, 6 d, 7 d	9		
	18 mm	5 d, 6 d, 7 d	9		
	25 mm	5 d, 6 d, 7 d	9		
	28 mm	5 d, 6 d, 7 d	9		
3	20 mm	4 d, 5 d, 6 d, 7 d, 8 d	15	Manufactured sand grout	
4	20 mm	8 d	3	Iron tailing sand grout	Eccentricity, Uniaxial tensile
	20 mm	8 d	3		Repeated tension and compression under high stress
	20 mm	8 d	3		Large-deformation reversed tension and compression
5	20 mm	8 d	3		Grout age 14 d

**Table 8:** Test parameters.

Group Number	Specimen Number	Rebar Diameter	Anchor Length (mm)	Number of Specimens	Types of Grout	Note
1	1-20-8	20 mm	8 d	3	Iron tailing sand grout	Uniaxial tensile
	1-20-7		7 d	3		
	1-20-6		6 d	3		
	1-20-5		5 d	3		
	1-20-4		4 d	3		
2	2-16-7	16 mm	7 d	7 d	Iron tailing sand grout	Uniaxial tensile
	2-16-6		6 d	6 d		
	2-16-5		5 d	5 d		
	2-18-7	18 mm	7 d	7 d	Iron tailing sand grout	Uniaxial tensile
	2-18-6		6 d	6 d		
	2-18-5		5 d	5 d		
	2-25-7	25 mm	7 d	7 d	Iron tailing sand grout	Uniaxial tensile
	2-25-6		6 d	6 d		
	2-25-5		5 d	5 d		
	2-28-7	28 mm	7 d	7 d	Iron tailing sand grout	Uniaxial tensile
	2-28-6		6 d	6 d		
	2-28-5		5 d	5 d		

**Table 9:** Unidirectional tensile test results with different anchorage lengths and key performance indicators.

Specimen No.	*P_y_* (kN)	*P_u_* (kN)	*f_u_*/*f_byk_*	*f_u_*/*f_buk_*	*τ_r_* (MPa)	*τ_max_* (MPa)	*S_y_* (mm)	*μ*_0_ (mm)	*A_sgt_* (%)	Sabotage Mode
1-20-8	141.8	187.2	1.49	1.10	/	>18.63	68.03	0.05	10.30	pull off
1-20-7	140.5	185.9	1.48	1.10	/	>21.14	63.05	0.08	7.31	pull off
1-20-6	139.6	184.9	1.47	1.09	/	>24.54	62.78	0.08	10.29	pull off
1-20-5	140.3	184.7	1.47	1.09	19.12	29.41	63.83	0.01	13.79	pull out
1-20-4	136.1	165.1	1.31	0.97	22.19	32.86	18.38	0.02	4.26	pull out

**Table 10:** Test parameters.

Group Number	Specimen Number	Rebar Diameter	Anchor Length (mm)	Number of Specimens	Types of Grout	Note
1	1-20-5(6, 7)	20 mm	5 d, 6 d, 7 d	9	Iron tailing sand grout	Uniaxial tensile
2	2-16-5(6, 7)	16 mm	5 d, 6 d, 7 d	9		
	2-18-5(6, 7)	18 mm	5 d, 6 d, 7 d	9		
	2-25-5(6, 7)	25 mm	5 d, 6 d, 7 d	9		
	2-28-5(6, 7)	28 mm	5 d, 6 d, 7 d	9		

**Table 11:** Uniaxial tensile tensile test results and key performance indicators for different rebar diameters.

Specimen No.	*P_y_* (kN)	*P_u_* (kN)	*f_u_*/*f_byk_*	*f_u_*/*f_buk_*	*τ_r_* (MPa)	*τ_max_* (MPa)	*S_y_* (mm)	*μ*_0_ (mm)	*A_sgt_* (%)	Sabotage Mode
2-16-5	86.5	118.2	1.47	1.09	/	>29.41	74.2	0.01	16.29	pull off
2-18-5	108.8	151.1	1.49	1.10	/	>29.70	58.7	0.01	10.30	pull off
1-20-5	140.3	184.7	1.47	1.09	19.12	29.41	63.83	0.01	13.79	pull out
2-25-5	211.2	287.4	1.47	1.09	15.17	29.29	30.31	0.04	/	pull out
2-28-5	271.2	364.7	1.48	1.10	18.37	29.63	59.46	0.12	/	pull out
2-16-6	91.4	124.0	1.54	1.14	/	>25.71	74.97	0.05	10.31	pull off
2-18-6	107.7	151.5	1.49	1.10	/	>24.82	64.44	0.06	13.30	pull off
1-20-6	139.6	184.9	1.47	1.09	/	>24.54	62.78	0.08	10.29	pull off
2-25-6	216.8	299.0	1.52	1.13	/	>25.39	79.67	0.09	/	pull off
2-28-6	271.7	366.4	1.49	1.10	/	>24.81	73.05	0.12	/	pull off
2-16-7	83.7	117.7	1.46	1.08	/	>20.92	59.06	0.04	8.29	pull off
2-18-7	108.1	151.2	1.49	1.10	/	>21.23	66.44	0.05	10.30	pull off
1-20-7	140.5	185.9	1.48	1.10	/	>21.14	63.05	0.08	7.31	pull off
2-25-7	211.3	294.1	1.50	1.11	/	>21.34	59.35	0.04	/	pull off
2-28-7	270.6	366.5	1.49	1.10	/	>21.27	71.80	0.08	/	pull off

**Table 12:** Test parameters.

Group Number	Specimen Number	Rebar Diameter	Anchor Length (mm)	Number of Specimens	Types of Grout	Note
1	1-20-8	20 mm	8 d	3	Iron tailing sand grout	Uniaxial tensile
	1-20-7		7 d	3		
	1-20-6		6 d	3		
	1-20-5		5 d	3		
	1-20-4		4 d	3		
3	3-20-8	20 mm	8 d	3	Manufactured sand grout	
	3-20-7		7 d	3		
	3-20-6		6 d	3		
	3-20-5		5 d	3		
	3-20-4		4 d	3		

**Table 13:** Uniaxial tensile test results and key performance indicators for different grout types.

Specimen No.	*P_y_* (kN)	*P_u_* (kN)	*f_u_/f_byk_*	*f_u_/f_buk_*	*τ_r_* (MPa)	*τ_max_* (MPa)	*S_y_* (mm)	*μ*_0_(mm)	*A_sgt_* (%)	Sabotage Mode
1-20-8	141.8	187.2	1.49	1.10	/	>18.63	68.03	0.05	10.30	pull off
3-20-8	132.0	190.5	1.52	1.12	/	>18.18	67.64	0.02	9.30	pull off
1-20-7	140.5	185.9	1.48	1.10	/	>21.14	63.05	0.08	7.31	pull off
3-20-7	137.7	183.9	1.46	1.08	/	>20.92	61.58	0.05	10.30	pull off
1-20-6	139.6	184.9	1.47	1.09	/	>24.54	62.78	0.08	10.29	pull off
3-20-6	137.7	185.8	1.48	1.09	/	>24.65	68.87	0.02	13.30	pull off
1-20-5	140.3	184.7	1.47	1.09	19.12	29.41	63.83	0.01	13.79	pull out
3-20-5	146.7	192.7	1.53	1.14	19.67	30.68	48.82	0.09	8.31	pull out
1-20-4	136.1	165.1	1.31	0.97	22.19	32.86	18.38	0.02	4.26	pull out
3-20-4	144.7	171.5	1.37	1.01	19.13	34.14	12.40	0.15	3.53	pull out

**Table 14:** Test parameters.

Group Number	Specimen Number	Rebar Diameter	Anchor Length (mm)	Number of Specimens	Types of Grout	Note
4	High stresses-20-8	20 mm	8 d	3	Iron tailing sand grout	Repeated tension and compression under high stress
	Large deformations-20-8	20 mm	8 d	3		Large-deformation reversed tension and compression

**Table 15:** Repeated tensile test results and key performance indicators.

Specimen Number	*P_y_* (kN)	*P_u_* (kN)	*f_u_/f_byk_*	*f_u_/f_buk_*	*S_y_* (mm)	Residual Distortion (mm)	*A_sgt_* (%)	Sabotage Mode
High stresses-20-8	127.4	181.7	1.45	1.07	44.55	*μ*_20_ = 0.230	8.29	pull off
Large deformations-20-8	139.9	183.2	1.46	1.08	53.21	*μ*_4_ = 0.046 *μ*_8_ = 0.109	6.29	pull off

**Table 16:** Test parameters.

Group Number	Specimen Number	Rebar Diameter	Anchor Length (mm)	Number of Specimens	Types of Grout	Note
1	20-8-28 d	20 mm	8 d	3	Iron tailing sand grout	28 d
5	20-8-14 d	20 mm	8 d	3		14 d
6	Eccentricity-20-8	20 mm	8 d	3		28 d Eccentricity

**Table 17:** Uniaxial tensile test results at different ages and key performance indicators.

Specimen Number	*P_y_* (kN)	*P_u_* (kN)	*f_u_/f_byk_*	*f_u_/f_buk_*	*τ_r_* (MPa)	*τ_max_* (MPa)	*S_y_* (mm)	*μ*_0_ (mm)	*A_sgt_* (%)	Sabotage Mode
20-8-28 d	141.8	187.2	1.49	1.10	/	>18.63	68.03	0.05	10.30	pull off
20-8-14 d	138.5	187.3	1.49	1.10	/	>18.93	53.14	0.06	7.30	pull off
Eccentricity-20-8	133.8	187.4	1.49	1.11	/	/	58.23	0.07	10.30	pull off

**Table 18:** Test parameters.

Specimen Number	Rebar Diameter	Anchor Length (mm)	Number of Specimens	Types of Grout	Note
5-20-8	20 mm	8 d	1	Iron tailing sand grout	Uniaxial stretching after attaching strain gauges
5-20-7		7 d	1		
5-20-6		6 d	1		
5-20-5		5 d	1		
5-20-4		4 d	1		

**Table 19:** Uniaxial tensile test results and key performance indicators for pasted strain gauge groups.

Specimen Number	*P_y_* (kN)	*P_u_* (kN)	*f_u_*/*f_byk_*	*f_u_*/*f_buk_*	*τ_r_* (MPa)	*τ_max_* (MPa)	*S_y_* (mm)	Sabotage Mode
5-20-8	139.6	184.7	1.47	1.09	/	>18.38	61.09	pull off
5-20-7	140.6	185.2	1.47	1.09	/	>21.06	62.97	pull off
5-20-6	133.7	184.4	1.47	1.09	12.79	24.47	46.45	pull out
5-20-5	139.8	174.0	1.39	1.03	13.92	27.71	20.24	pull out
5-20-4	142.9	169.3	1.35	1.00	20.49	33.70	18.14	pull out

## Data Availability

The data supporting this study will be made available upon request.
